# Dissecting the Functional Role of the N-Terminal Domain of the Human Small Heat Shock Protein HSPB6

**DOI:** 10.1371/journal.pone.0105892

**Published:** 2014-08-26

**Authors:** Michelle Heirbaut, Steven Beelen, Sergei V. Strelkov, Stephen D. Weeks

**Affiliations:** Laboratory for Biocrystallography, Department of Pharmaceutical and Pharmacological Sciences, KU Leuven, Belgium; UMCG, Netherlands

## Abstract

HSPB6 is a member of the human small heat shock protein (sHSP) family, a conserved group of molecular chaperones that bind partially unfolded proteins and prevent them from aggregating. In vertebrate sHSPs the poorly structured N-terminal domain has been implicated in both chaperone activity and the formation of higher-order oligomers. These two functionally important properties are likely intertwined at the sequence level, complicating attempts to delineate the regions that define them. Differing from the prototypical α-crystallins human HSPB6 has been shown to only form dimers in solution making it more amendable to explore the determinants of chaperoning activity alone. Using a systematic and iterative deletion strategy, we have extensively investigated the role of the N-terminal domain on the chaperone activity of this sHSP. As determined by size-exclusion chromatography and small-angle X-ray scattering, most mutants had a dimeric structure closely resembling that of wild-type HSPB6. The chaperone-like activity was tested using three different substrates, whereby no single truncation, except for complete removal of the N-terminal domain, showed full loss of activity, pointing to the presence of multiple sites for binding unfolding proteins. Intriguingly, we found that the stretch encompassing residues 31 to 35, which is nearly fully conserved across vertebrate sHSPs, acts as a negative regulator of activity, as its deletion greatly enhanced chaperoning capability. Further single point mutational analysis revealed an interplay between the highly conserved residues Q31 and F33 in fine-tuning its function.

## Introduction

Acting as ATP-independent molecular chaperones, small heat shock proteins (sHSPs) play an important role in protein house-keeping [1]–[3]. These proteins, found in all kingdoms of life, function by binding partially unfolded protein species keeping them in a soluble state [4]. As their name suggests, their expression levels respond to environmental stress but some members are also produced constitutively at high levels [5]. sHSPs are regarded as the first-line of defense for a cell dealing with aberrant protein species and work in concert with the other chaperone families to maintain cellular proteostasis [6]–[8]. Besides their function in the protein quality control network, members of this family also display distinct roles in a variety of cellular pathways and biological processes [9].

All sHSPs share the same structural arrangement consisting of a conserved region of approximately 90 residues, called the α-crystallin domain (ACD), flanked by unstructured N- and C-terminal arms that vary in sequence and length [10]. The ACD has a β-sandwich fold that is capable of dimerization. These ACD-mediated dimers are considered to be the basic building blocks of the higher-order oligomers generally associated with this family of proteins [1], [9], [11]. While predicted to be unstructured, the N- and C-terminal domains are necessary for oligomer formation [12]–[14]. In vertebrates these oligomeric assemblies are typically polydisperse in subunit number and shape, and at physiological temperatures show a high turnover of the individual subunits [15]–[18]. The exact mechanism of chaperone action is not fully understood, however the general hypothesis is that sHSPs can bind unfolding proteins via exposed hydrophobic surfaces, thereby creating a “kinetic partitioning” [1] where the substrate binds the hydrophobic sites on the sHSP rather than interacting with other metastable species [1], [11]. Some models have proposed a mechanism where the recognition of partially unfolded proteins or heat stress leads to the dissociation of the oligomers, releasing dimers that can capture non-native proteins. These smaller subunits then reassemble into the oligomer forming larger sHSP:client protein complexes [11].

**Figure 1:**
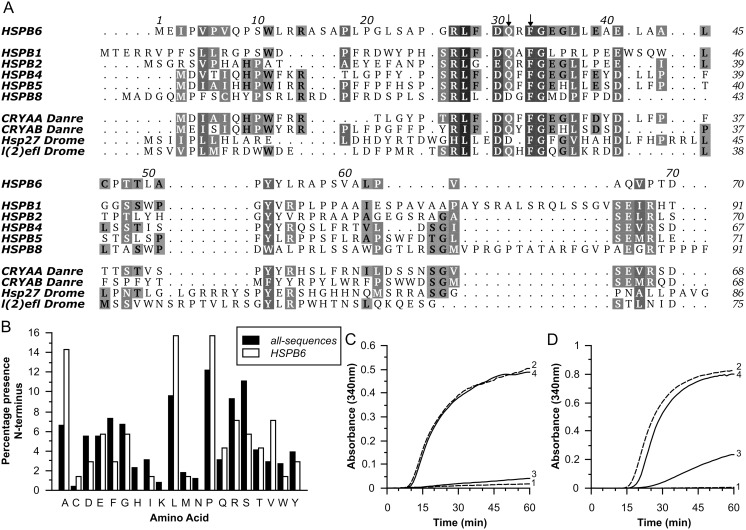
Properties of the N-terminal domain of HSPB6. (A) Multiple sequence alignment of the NTD of HSPB6 with other human sHSPs, the α-crystallins from *Danio rerio* (CRYAA and CRYAB) and two related proteins from *Drosophila melanogaster* (l(2)_efl and Hsp27). The two highly conserved residues that are mutated in this work are indicated by arrowheads. The alignment was made using Aline [54]. Uniprot accession numbers: HSPB1 (Hsp27): P04792, HSPB2: Q16082, HSPB4 (CRYAA): P02489, HSPB5 (CRYAB): P02511, HSPB6: O14558, HSPB8: Q9UJY1, CRYAA_Danre: Q8UUZ6, CRYAB_Danre: Q9PUR2, Hsp27_Drome: P02518, l(2)efl_Drome: P82147. (B) Amino acid composition within the N-terminus of HSPB1, HSPB2, HSPB4, HSPB5, HSPB6 and HSPB8 and orthologues in different species (*H. sapiens*, *B. taurus*, *M. musculus*, *X. leavis* or *tropicalis*, *G. gallus* and *D. rerio*). The average amino acid composition is shown as white bars, HSPB6 amino acid composition is shown as black bars. (C–D) Chaperone-like activity of human HSPB6 and the N-terminal deletion construct (ΔN) using insulin (C) and yADH (D) as substrates. The monomer mass molar ratios of substrate:sHSP were 1∶0.2 and 1∶2 respectively, and were the same as used in further experiments. Curve 1, substrate alone; 2, substrate + DTT; 3, with addition of HSPB6; 4, with addition of ΔN.

A number of studies have been conducted to pinpoint the sequence-specific epitopes that define sHSP chaperone activity, and the majority support a central role for the N-terminal domain (NTD). These analyses have primarily focused on the canonical members of the sHSP-family: the α-crystallins and HSPB1 [12], [13], [19]–[21]. Comparative analysis suggests that different regions of the NTD are involved in chaperone activity, with some studies reporting contradictory results [12], [20], [22]. These ambiguities can be ascribed to the species origin of the sHSP as the NTD is often purported to be poorly conserved. However sequence analysis of vertebrate sHSPs show that orthologues from distantly related species share high similarity (Fig. S1). Although paralogues within a single species demonstrate more sequence divergence, the NTD contains a highly conserved region (Fig. 1A and Fig. S1) and comparative genome analysis suggests an overall strong amino acid bias amongst vertebrate sHSPs (Fig. 1B) [10]. Likely more crucial to the observed differences is the overlapping role of the NTD in both chaperoning and higher-order assembly [12], [13], [19], [22]. This dual function makes it difficult to separate the effect of mutation on either property alone.

In this study we have determined the specific regions required for chaperoning in human HSPB6 (Hsp20). Rat HSPB6 was originally described to be a poor chaperone [23], but more recent studies have shown that the human orthologue has equivalent activity to αB-crystallin [24]. Importantly, in solution, human HSPB6 only forms dimers [24], [25] that possibly represent the basic chaperoning active subspecies of all sHSP family members. Recent efforts have led to a partial atomic structure of fragments of the rat and human HSPB6, and by using a combination of crystallography and small-angle X-ray scattering (SAXS), a solution model for the full-length protein composed of the ACD-based dimer with exposed but compact N-termini has been proposed [25], [26]. Although the protein shows the propensity to transiently self-associate, even at high concentrations the formation of a higher-order oligomeric assembly has not been observed [25], [27], [28]. This inability to form oligomers occurs despite the NTD showing similar sequence length and properties to the α-crystallins (Fig. 1A and Fig. S1). HSPB6 is thus an excellent candidate for studies on elucidating the role of the NTD in defining chaperone-like activity.

## Experimental Procedures

### Mutagenesis and cloning

**Figure 2:**
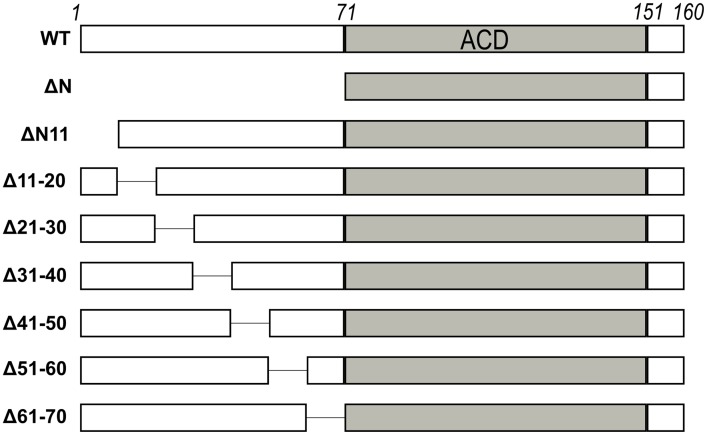
N-terminal 10-residue deletion mutants. Cartoon of the different HSPB6 constructs used in this work. The α-crystallin domain (ACD) is shown in grey. The numbers at the top of the cartoon correspond to the residue number at domain boundaries.

The previously described small ubiquitin modifier (SUMO)-fusion of HSPB6 [28] was used as a template for the generation of the different deletion constructs and point mutants. All 10 amino acid N-terminal deletions (Fig. 2A) were generated using whole plasmid amplification, where one primer contained an extension of sequence complementary to the other (Table S1). Point mutations were created using Quickchange mutagenesis. DpnI-treated PCR products were transformed into *E. coli* NEB5α (New England Biolabs) and positive clones were verified by sequencing. HSPB6 variants ΔN, ΔN11 and HSPB4 were created using the In-Fusion cloning kit (Clontech) into pETHSUL [29]. The template sequence for HSPB4 amplification was pANT7-cGST. HSPB4 obtained from the DNASU plasmid repository [30]. The full-length HSPB1 expression construct was described previously [28]. All constructs were designed such that, upon cleavage of the linearly fused SUMO chimera with recombinant SUMO-hydrolase, no additional non-native residues would be present on the target protein.

### Expression and purification

All constructs were over-expressed in the *E. coli* NiCo21 (DE3) strain (New England Biolabs) containing pRARE2 (EMD Biosciences). Transformed clones were cultured in ZYP-5052 auto-inducing medium [31] using described conditions [28]. Lysis, clarification and subtractive immobilized metal affinity chromatography (IMAC) of the SUMO-fusions were carried out as reported previously [29]. Following the initial isolation steps all proteins were dialyzed to 20 mM Tris-HCl pH 7.0, containing 2.5 mM DTT and 10% w/v glycerol and further purified by anion-exchange chromatography loading the sample onto a HiTrap HP Q column (GE Healthcare). The bound protein was eluted using a linear gradient from 0 to 200 mM NaCl in 20 mM Tris-HCl, pH 7.0 and 2.5 mM DTT over 20 column volumes. Pooled fractions were concentrated and additionally purified by size exclusion chromatography (SEC) using a Superdex 200 16/60 PG column (GE Healthcare) equilibrated in 50 mM sodium phosphate pH 7.5, 100 mM NaCl. For HSPB1 and HSPB4 the same protocol was followed except for the final SEC step, which was performed in the same buffer complemented with 2.5 mM DTT. All purification steps were carried out at 4°C. Protein fractions were checked by SDS-PAGE, pure samples were pooled together and concentrated to approximately 20 mg/ml. Aliquots of purified protein were flash-frozen in liquid nitrogen and stored at −80°C until required.

### Analytical SEC

The various HSPB6 constructs were examined by analytical SEC at 4°C. 100 µl samples of each construct at a concentration of 2.5, 5 and 10 mg/ml were loaded onto a Superdex 200 10/300 GL column (GE Healthcare) pre-equilibrated in 25 mM HEPES pH 7.5, 150 mM NaCl and 2.5 mM DTT. The frozen protein aliquots were thawed and diluted in 50 mM sodium phosphate, 100 mM NaCl, pH 7.5 complemented with 10 mM DTT. After incubation for 20 minutes on ice, samples were centrifuged at 4°C for 10 min at 14200 g prior to loading. The ΔN11-construct of HSPB6 was not analyzed on SEC as this construct could not be concentrated above 5 mg/ml. Column calibration was performed using the Molecular Weight Calibration Kit from GE Healthcare.

### Small-angle X-ray studies

SAXS data were collected at the SWING beamline (Soleil Synchrotron, Saint-Aubin, France) with an inline HPLC system. The purified concentrated proteins were thawed and their concentrations were measured with a Nanodrop spectrophotometer (Thermo Scientific) using their theoretical specific absorption coefficient. A 200 µl sample was prepared containing 5 mg/ml of HSPB6 by diluting the concentrated sample with column buffer (20 mM HEPES, 150 mM NaCl, pH 7.4 and 2.5 mM DTT). The other constructs were prepared in the same manner to yield equivalent molarity. Immediately prior to SAXS measurement, the samples were centrifuged at 14200 g at 4°C for 10 min. 75 µl of each sample was loaded onto a Shodex KW404-4F column pre-equilibrated in the column buffer with a flow-rate of 0.2 ml/min at 20°C, for the ΔN11-construct 111 µl of a 3.37 mg/ml solution was loaded as this protein could not be concentrated above this value. During each HPLC run, SAXS data for both buffer and protein were collected. For the buffer 100 frames were collected starting 5 min after injection with an exposure time of 0.75 s and dead time of 1 s. 250 frames were collected for the protein starting after 16 min using the same exposure and dead times. Buffer frames were processed, averaged and subtracted from each protein frame using Foxtrot (software available on site at SWING-beamline). The radius of gyration (R_g_) and forward scattering (I_0_) were calculated for each buffer subtracted protein curve using AutoRg [32], with data that had been averaged using a ten frame moving average algorithm to improve the signal-to-noise ratio. For each sample ten scattering curves, collected around the peak maximum, were averaged and then scaled to the curve with the maximum forward scattering value (I_0_). These averaged curves were used for further analysis. The R_g_ and I_0_ were calculated in PRIMUS using both AutoRg and GNOM and the volumes of the scattering particles were determined using DATPOROD, available in the Atsas-package [32]. The molecular weight of each protein was determined on an absolute scale using the I_0_ of water [33], as well as based on the Porod volume using a protein density of 0.588 Da/Å^3^ [32].

### Bis-ANS binding studies

A stock solution of bis-ANS (4,4′-Dianilino-1,1′-binaphthyl-5,5′-disulfonic acid, Sigma) was prepared in 95% ethanol and the concentration was determined by measuring the absorbance of a 1∶10 dilution in water at 385 nm using an extinction coefficient of 16790 M^−1^ cm^−1^. Serial dilutions of the bis-ANS stock, with a ten-fold higher concentration than final target value were freshly prepared in 95% ethanol to ensure that the final amount of ethanol present in each sample remained constant (10% of final volume). The binding assay was performed at room temperature using 0.04 mg/ml (equivalent to 2.5 µM) of each protein construct diluted in 50 mM sodium phosphate pH 7.5, 100 mM NaCl and 5 mM DTT and mixed with increasing amounts of bis-ANS with a total volume of 150 µl. Fluorescence was measured in a 96-well black, flat-bottom polypropylene plate (Greiner BioOne) on an Infinite M200 fluorescence plate reader (Tecan Instruments). The solutions were excited at 390 nm and emission intensities were measured at 490 nm, the photomultiplier settings were set at an optimal gain to ensure highest sensitivity. All values are measured in arbitrary units and were normalized to those determined for bis-ANS binding to HSPB6. All values were corrected for the fluorescence from bis-ANS alone and each measurement was performed in triplicate.

### Chaperone assays

Human recombinant insulin (Sigma) was dissolved in 2.5% acetic acid, hen egg white lysozyme (HEWL, Sigma) and recombinant yeast alcohol dehydrogenase (yADH, Sigma) were dissolved 50 mM sodium phosphate, 100 mM NaCl, pH 7.5 at a concentration of approximately 5 to 10 mg/ml. All substrate proteins were dialyzed overnight at 4°C using SpectraPor Dialysis membrane with a 3 kDa molecular weight cut-off (MWCO, SpectrumLabs) into 50 mM sodium phosphate, 100 mM NaCl, pH 7.5. HSPB6 and its mutants were thawed and if necessary also dialyzed overnight at 4°C against the same buffer using Slide-A-Lyzer mini dialysis unit with a 3 kDa MWCO (Thermo Scientific). The concentration of each protein, including the substrates was determined using a Nanodrop spectrophotometer (Thermo Scientific). The substrate protein was added to assay buffer to yield a final concentration of 0.25 mg/ml and sHSP was added to give different molar ratios (substrate:sHSP). The mixture containing substrate and sHSP was allowed to incubate for 20 minutes at room temperature. Aggregation of insulin and HEWL was induced by the addition of 10 mM of DTT (final concentration) in 50 mM sodium phosphate and 100 mM NaCl to the pre-incubated assay mixture. To induce aggregation of yADH, the sample was heated to 42°C in presence of 20 mM DTT and 2 mM EDTA. All assays were performed in a final volume of 180 µl in a 96-well, flat-bottom, half-area UV-transparent plate (UV-Star, Greiner Bio-one). Aggregation was followed by monitoring light scattering at 340 nm in an ELx808IU plate reader (Biotek) at 37°C (insulin and lysozyme) or 42°C (yADH) for 90 min. The optical density was measured each minute and blanked to the buffer. The chaperone activity was defined as the percentage protection, where the absorbance at 340 nm of substrate alone (without DTT) was taken as 100%, and the absorbance of the substrate with DTT after 90 minutes as 0%. Each condition was performed in triplicate.

### Dynamic light scattering studies

DLS measurements were performed on HSPB6 alone and on the mixture containing insulin and HSPB6 after DTT-induced aggregation. For HSPB6, a 1 mg/ml solution was made in 50 mM sodium phosphate, 100 mM NaCl and 2.5 mM DTT, pH 7.5. For the mixture of insulin:sHSP, 0.25 mg/ml insulin was incubated with wild type HSPB6 or Δ31–40 in the same monomer molar ratio as in the chaperone assays. After incubation at 37°C for 90 min, the scattered light was measured using a Zetasizer µV (Malvern Instruments) in a 12 µl cuvette, with automatic measurement duration. Data analysis was performed using the Zetasizer Software (Malvern Instruments) and for all samples a refractive index of 1.450 and a viscosity of 0.7451 cP were used. The intensity-weighted value of the mean molecular size (Z-average) was determined by single cumulant fitting of the decay function, which yielded an estimate of the hydrodynamic radius (R_h_). The molecular weight (M_w_) was estimated from the hydrodynamic radius using the equation:
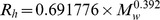
where R_h_ is in nm and M_w_ is in KDa [34].

## Results

### Expression and purification of HSPB6 deletion mutants

The NTD of vertebrate sHSPs has previously been described as a determinant of chaperoning activity. To verify the importance of this region for human HSPB6– corresponding to the first 70 amino acids (Fig. 1A and Fig. S1) – we have cloned and expressed a construct termed ΔN, which was completely missing this region. At equivalent monomer molar concentrations to the full-length protein, ΔN failed to prevent DTT-induced aggregation of human insulin and yeast alcohol dehydrogenase (yADH) (Fig. 1C and D) despite showing similar thermal stability as the full-length protein (Fig. S5). Thus, the NTD of HSPB6 is essential for chaperone activity at least for the two substrates tested.

To further delineate the sequence motifs within the NTD that are important for activity, we cloned and successfully expressed seven additional HSPB6 constructs, with systematic 10 amino acid deletions covering the whole NTD (Fig. 2A). All constructs, except ΔN10, gave suitable yields of pure protein for biochemical and biophysical characterization (Fig. S2). Showing equivalent solubility to the wild-type protein, all could be readily concentrated above 25 mg/ml.

In the case of the ΔN10 construct, the SUMO fusion expressed well and could be purified by IMAC, but the protein precipitated during digestion of the fusion with recombinant SUMO hydrolase. As this construct, after proteolytic cleavage, begins with the sole tryptophan found in HSPB6 (Fig. 1A), we created a longer truncation termed ΔN11, that removed this hydrophobic residue. Even though this truncation showed some precipitation upon concentration, it did display higher solubility than ΔN10, and could be concentrated to approximately 4 mg/ml. The poor solubility of these two constructs contrasts markedly with the behavior of the full-length protein and the other deletion variants. Interestingly, in a previous study [25] an even longer N-terminal deletion construct, where the first 23 amino acids of HSPB6 were deleted showed solubility equivalent to the full-length protein. This, in combination with the fact that the ΔN11–20 also behaved well, suggests that the poor solubility observed for both ΔN10 and ΔN11 can be ascribed to residues 12 to 22. Surprisingly this region contains two arginines and a limited number of small hydrophobic residues that alone cannot explain the observed reduction in solubility (Fig. 1A).

### Biophysical characterization of HSPB6 and truncations

**Figure 3:**
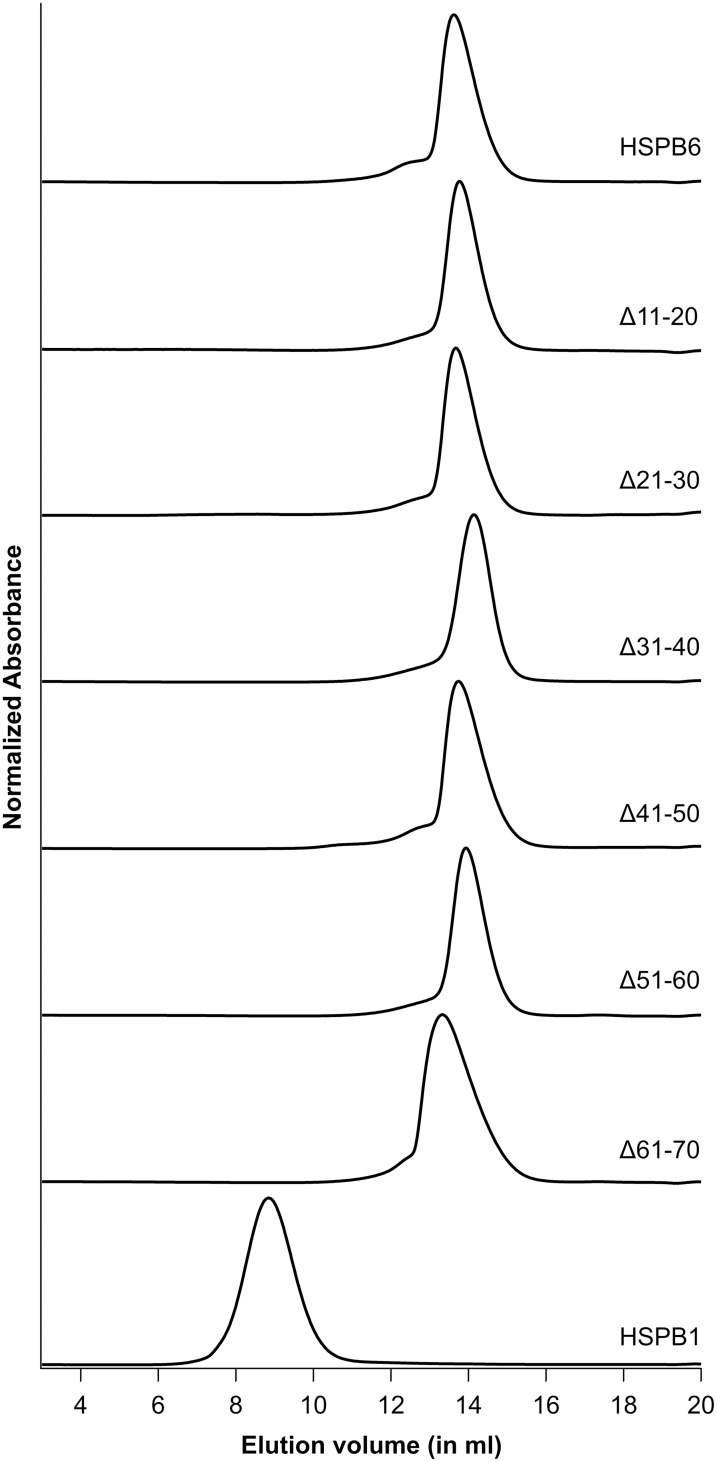
Analytical SEC of the HSPB6 10-residue deletion mutants and HSPB1. 100 µl of each protein at 10 mg/ml was loaded on a Superdex 200 10/300 column at 4°C equilibrated in 25 mM HEPES, 150 mM NaCl and 2.5 mM DTT at pH 7.5.

The oligomeric size and the self-association properties of the various HSPB6 constructs were assessed by SEC using a Superdex 200 GL column. For the wild type protein and all 10-residue deletions, the SEC profile contained a single elution peak, which corresponded to a radically smaller species compared to the HSPB1 oligomer (apparent mass 800 kDa, Fig. 3).

**Figure 4:**
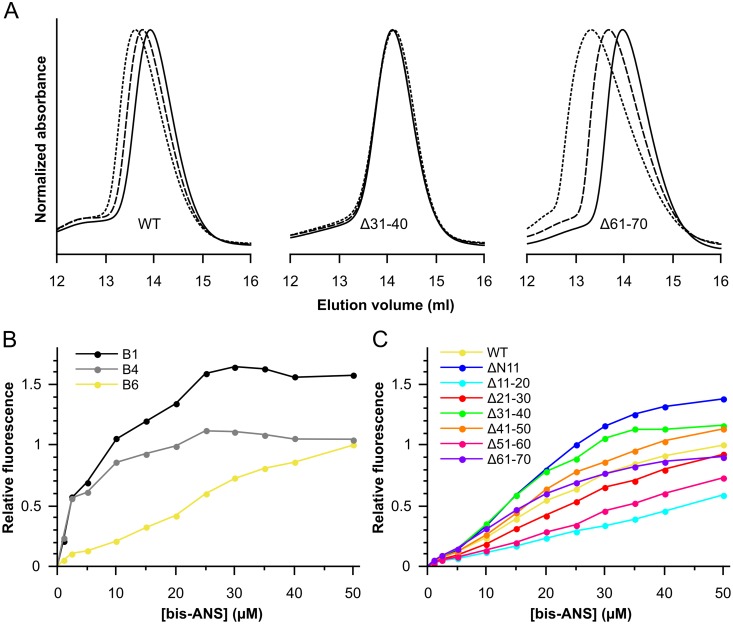
Solution properties of the HSPB6 10-residue deletion mutants. (A) Size-exclusion chromatography profiles of wild-type HSPB6, Δ31–40 and Δ61–70. 100 µl of protein at three different concentrations was loaded on a Superdex 200 10/300 GL column. 2.5 mg/ml is depicted as a solid line, 5 mg/ml as a dashed line and 10 mg/ml as a dotted line. The absorbance was normalized to the maximum absorbance for each curve. (B) Bis-ANS titration curves for HSPB1, HSPB6 and HSPB4. 2.5 µM protein was mixed with increasing concentrations of bis-ANS and fluorescence intensity was recorded at 490 nm using an excitation wavelength of 390 nm. HSPB6 is shown in yellow, HSPB1 in black and HSPB4 in grey. (C) Bis-ANS titration curves for all constructs, the same setup as for HSPB1 and HSPB4 was used. HSPB6 is shown in yellow, ΔN11 in blue, Δ11–20 in cyan, Δ21–30 in red, Δ31–40 in green, Δ41–50 in orange, Δ51–60 in pink and Δ61–70 in violet.

Notably the elution peak for HSPB6 shifted towards smaller elution volumes upon increase of the loaded sample concentration, corresponding to an increase of the apparent molecular weight (Fig. 4A, Table 1) [25], [27]. This shift in the elution volume cannot be explained by the formation of larger assemblies, as one would then expect additional distinct peaks in the chromatogram, but rather by non-ideal associative behavior resulting from attractive forces between the individual protein molecules. With the exception of Δ31–40, all other 10-residue deletion constructs also displayed the capacity to self-associate (Table 1). For the former construct, the peak maxima position remained the same for all three concentrations tested (Fig. 4A).

**Table 1:** Biophysical properties of wild-type HSPB6 and its mutants.

Construct	Concentration dependenceof apparent MW^a^(kDa/(mg/ml))	Hydropathy ofthe NTD^b^
WT	1.51	0.167
ΔN11	N/A	0.137
Δ11–20	0.81	0.209
Δ21–30	1.29	0.172
Δ31–40	–0.14	0.194
Δ41–50	1.92	0.128
Δ51–60	0.31	0.171
Δ61–70	3.40	0.147
Δ31–35	–0.01	0.218
Δ36–40	1.80	0.142
Q31A	1.10	0.188
Q31L	1.87	0.194
F33A	–0.21	0.159
F33Y	0.42	0.154

a Slope of a linear fit of the calculated MW (determined from calibration with the Molecular Weight kit from GE Healthcare) at three different concentrations.

b Calculated as the average over the NTD using the hydrophobicity scale according to Eisenberg *et al.* [53].

Despite the non-ideal behavior, the elution volumes for all constructs at the lowest loaded concentration were very similar, suggesting the same molecular weight (Fig. 3). The Δ61–70 construct however, was more polydisperse (based on peak width at half height), slightly bigger when compared to the wild-type protein at the lowest concentration, and showed a larger increase in size upon increasing concentration (Fig. 4A and Table 1). For this single construct the more pronounced shift in size suggests the possibility of the presence of higher-order structures, albeit still much smaller than the HSPB1 oligomers (Fig. 3).

**Figure 5:**
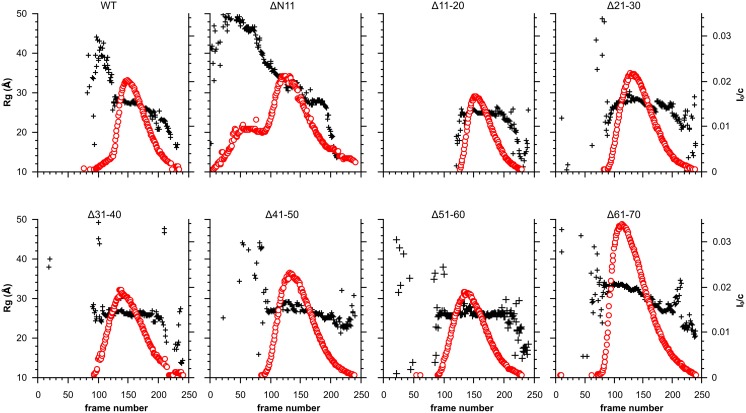
SAXS analysis of the HSPB6 10-residue deletion mutants. For each construct, 250 frames were collected during elution from a Shodex KW-404F column. The radius of gyration (R_g_) and forward scattering (I_0_) were calculated at each measured point using buffer subtracted scattering curves that had been averaged by a ten-frame moving average algorithm to improve the signal-to-noise. The forward scattering values are normalized by dividing the values by the concentration of the sample at the peak maximum. The R_g_ is plotted as a black cross for each construct and scaled on the left axis, the I_0_ is shown as a red circle and scaled on the right axis.

To further investigate the effect of the truncations on the structure of HSPB6, small angle X-ray scattering experiments were performed on a set-up with an inline HPLC-system. For the wild-type protein and all truncations, 250 scattering frames were collected at a single loaded concentration during peak elution from the column. Guinier analysis was performed on each buffer subtracted, radially averaged frame to calculate the radius of gyration (R_g_) and forward scattering (I_0_). These values were plotted against the frame number to create a SAXS-based elution profile (Fig. 5). The normalized I_0_ values, determined on an absolute scale by calibration with water, correspond to the mass of the scattering species. For all constructs, except Δ61–70, the normalized I_0_ at the peak maximum was close to that of the full-length protein with a value of 0.02. Calculation of the molecular weight for each construct, either by using the measured I_0_ values at the peak maxima or based on determination of the Porod volume from the averaged SAXS curve at the same point, consistently yielded a mass close to a dimer for all samples, with the exception of Δ61–70 (Table 2). This latter construct had a calculated molecular weight closer to a tetramer, agreeing with the SEC analysis that it forms slightly larger assemblies. For full-length HSPB6 the SAXS-based R_g_ values vary from approximately 28 Å to 25 Å for the frames that have an I_0_ value above the peak half height, with an R_g_ of 27.5 Å at the peak maximum (Table 2). Similarly, the majority of the constructs had an R_g_ of approximately 27 Å at their peak maxima, suggesting there are no significant changes in shape when compared to the full-length protein (Fig. 5 and Table 2). The biggest R_g_ difference across the elution peak was observed for ΔN11, with a decrease from 36 to 28 Å. This construct also showed a double peak based on the intensity at the zero scattering angle with a continually decreasing R_g_, confirming the aggregating behavior of this construct, nonetheless the I_0_ and calculated R_g_ of the soluble fraction still suggest a dimeric structure for this construct. The calculated R_g_ for Δ61–70 of 33 Å is also bigger than that of the full-length HSPB6, although the difference across the peak for the frames with an I_0_-value above half-maxima was the same. This again supports the interpretation that this construct forms slightly larger oligomeric assemblies at an equivalent concentration to the others.

**Table 2:** SAXS data analysis.

Construct	Estimated MW (kDa)		Calculated numberof subunits	R_g_ (Å)	Concentration(mg/ml)^a^
	based on I_0_ of water	based on Porod volume			
WT	31.4	38.1	2.2	27.5±1.8	0.61
ΔN11	31.2	39.9	2.5	31.9±2.8	0.65
Δ11–20	25.7	29.5	1.8	25.2±1.1	0.47
Δ21–30	32.8	35.9	2.2	28.7±0.7	0.89
Δ31–40	28.2	32.9	2.0	26.7±2.2	0.79
Δ41–50	34.8	36.6	2.3	28.2±0.7	0.88
Δ51–60	28.3	30.8	1.9	27.3±1.3	0.87
Δ61–70	51.6	56.0	3.5	33.4±0.5	0.77
Δ31–35	30.9	35.3	2.2	27.4±1.3	0.97
Q31A	67.4	71.2	4.2	37.5±0.8	1.37
Q31L	64.7	72.9	4.3	38.0±1.2	1.31
F33A	29.4	38.4	2.3	30.2±1.0	2.32
F33Y	36.8	42.6	1.9	30.9±1.6	1.77

a The concentration for each construct at the peak maximum was calculated using the maximum absorbance at 280 nm as it eluted from the column and their calculated absorbance coefficients.

Comparison of the shape of the averaged SAXS curves at the elution peak maxima (Fig. S3, top row) suggested that none of the truncations underwent major structural changes relative to the full-length protein. For all constructs analysis of the dimensionless Kratky plots (Fig. S3, bottom row) showed the same features as present for the wild type protein: a parabolic curve, signifying the presence of a folded domain, followed by a slightly elevated baseline at higher q.Rg values. In almost all cases the position and height of the peak maxima (2.2, 1.3) was similar. These values are higher than that expected for an ideal sphere (√3, 1.1 and Fig. S3), suggesting a more extended structure [35]. This data is in good agreement with a recently proposed model of HSPB6 composed of the core ACD dimer with an ensemble of partially extended N-terminal arms [25], and further supports the conclusion that the deletions do not affect the overall structure of the protein. Only ΔN11 showed a maximum at higher q•R_g_, when compared to full-length HSPB6, which could be anticipated from the SEC data and R_g_ calculation, and again might be a result from its aggregating propensity.

Finally the same conclusions can be inferred by the pair distribution functions (Fig. S3, middle row). All plots showed an asymmetric peak, suggesting a slightly elongated structure [36], with most distances centered at 27 Å. With the exception of ΔN11 and Δ61–70, the truncations all have a D_max_ of approximately 95 Å. These values are similar to the wild type protein again suggesting no major changes in the structure for the majority of constructs.

### Bis-ANS binding

To study the relative hydrophobicity of the different HSPB6 truncations, and its possible correlation with chaperone activity, the binding properties of the environmental probe bis-ANS were investigated. The full-length protein showed a linear increase in fluorescence with increasing bis-ANS concentration but no obvious saturation in association over the concentration range tested (Fig. 4B and C). This behavior contrasts with human HSPB1 and HSPB4 - two sHSPs that form large oligomeric species - both of which show a typical hyperbolic curve (Fig. 4B). The difference can be explained by the fact that the large oligomers of HSPB1 and HSPB4 contain an accessible solvent-free hydrophobic core with high affinity for bis-ANS. On the contrary the HSPB6 dimers bind bis-ANS with a reduced affinity due to the absence of such a structure. The same phenomenon has been observed for human HSPB8, a potent chaperone which also only forms small oligomeric species in solution [37].

For all the truncations the bis-ANS titration profiles were more similar to the full-length HSPB6 than that of HSPB1 and HSPB4 (Fig. 4B and C), suggesting that none of deletions resulted in a large alteration in affinity. In comparison to the other constructs Δ11–20 and Δ51–60 demonstrated a lower final fluorescence. Although this indicates a reduced hydrophobicity, the calculated hydropathy of the NTD of these two deletions was conversely higher than that of the full-length protein (Table 1), due to the removal of arginine residues in both cases (Fig. 1A). Even though bis-ANS is a probe for a hydrophobic environment, it has been recognized that the negatively charged sulfate groups might interact with arginines on proteins that are in close proximity to hydrophobic patches and result in enhanced bis-ANS fluorescence [38]. Thus the reduction in fluorescence in these two deletion constructs may be explained purely by the removal of these residues.

Three constructs ΔN11, Δ31–40 and Δ61–70 do appear to reach a plateau with increasing bis-ANS concentration suggesting an increased affinity for this probe when compared to the full-length protein. For ΔN11 and Δ61–70 this is likely due to the higher propensity of the former to aggregate and the latter to reversibly associate in solution (Fig. 4 and 5), both phenomena possibly yielding structures containing a more apolar environment. In the case of Δ31–40, which showed no concentration dependent effect on elution volume (Fig. 4A), this can be explained by the loss of a number of charged residues in this region (Fig. 1A).

### Chaperone activity of HSPB6 and deletions

**Figure 6:**
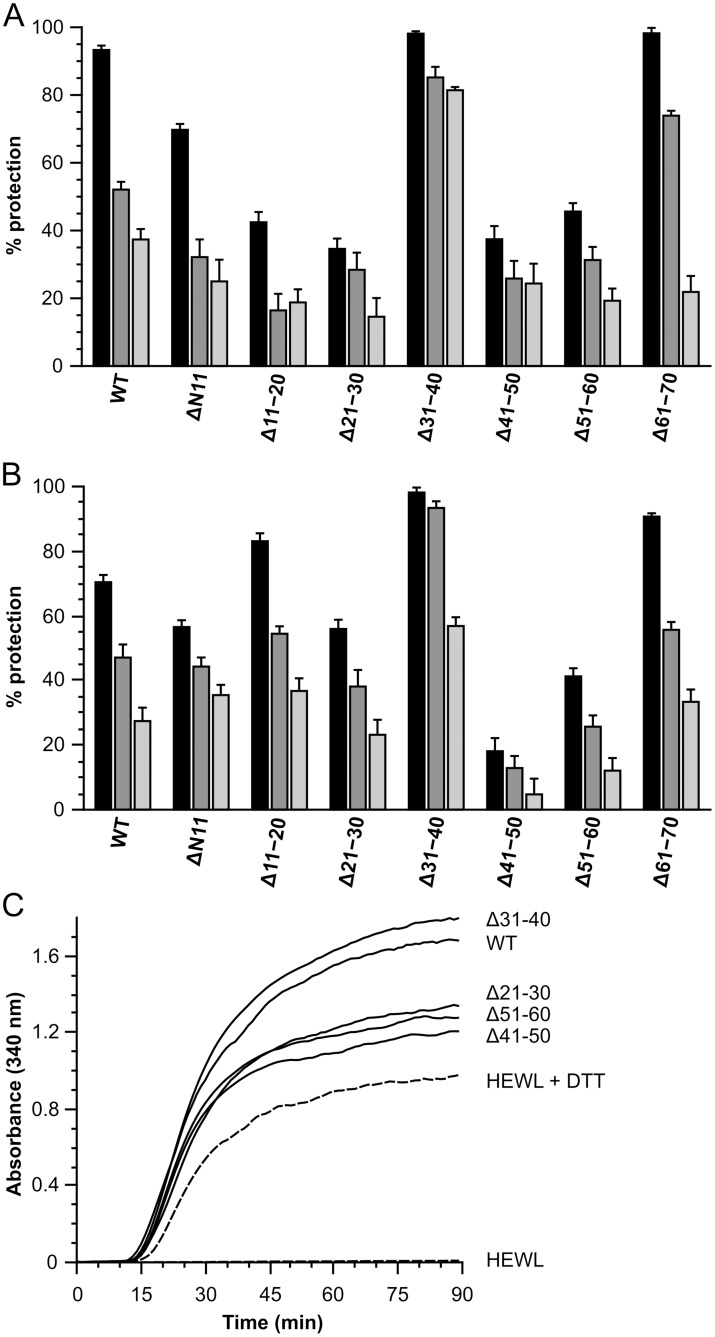
Chaperone-like activity of HSPB6 and the 10-residue deletion mutants. Aggregation was monitored by following the absorbance at 340 nm for 90 min. The percentage protection for each construct was calculated as described in the Materials and Methods. (A) 0.25 mg/ml insulin used as a substrate. Aggregation was induced by adding a final concentration of 10 mM DTT prior to continuous measurement at 37°C. The ratios of substrate to sHSP are 1∶0.2 (black), 1∶0.1 (gray) and 1∶0.05 (light gray) (B) 0.25 mg/ml yADH used as a substrate. Aggregation was induced by adding 20 mM DTT and 2 mM EDTA (final concentration) prior to measurement at 42°C. The monomer mass molar ratios of substrate to sHSP were 1∶2 (black), 1∶1 (gray) and 1∶0.5 (light gray). (C) DTT-induced aggregation of HEWL in absence and presence of HSPB6 and the truncations. For clarity not all constructs are shown. The monomer mass molar ratio of HEWL:sHSP was 1∶2.

Concluding that deletion mutants did not result in a major structural change, the chaperone activity of each was compared to full-length HSPB6 using insulin, yADH and hen egg white lysozyme as substrates (Fig. 6 and Fig. S5). For all three proteins, aggregation could be readily induced by reduction of the disulfide bonds. Under the same conditions all HSPB6 constructs were stable on their own (Fig. S5, J–K). For ΔN11 the chaperone-like activity was determined despite its propensity to aggregate at concentrations above 5 mg/ml. The concentrations used in the assays were below the solubility limit for this construct, and results from our biophysical characterization show that the soluble fraction has a similar structure and oligomeric state as HSPB6.

As opposed to the full NTD deletion, the smaller deletions all showed some capacity to protect insulin from aggregation, even at a four-fold lower concentration (Fig. 6A). Both ΔN11 and Δ61–70, the deletion mutants corresponding to the two ends of the NTD, demonstrate activities similar to the WT protein (Fig. 6A). Four constructs Δ11–20, Δ21–30, Δ41–50 and Δ51–60 showed, on average, a 50% decrease in the ability to protect insulin against aggregation at all substrate:sHSP ratios tested (Fig. 6A). Intriguingly Δ31–40, the region found in center of the NTD, demonstrated nearly 100% protection at all substrate:chaperone ratios tested, thus displaying an approximate five-fold increase in chaperone activity relative to wild-type HSPB6. For all constructs the change in the protective capacity manifested itself purely in the final value of the absorbing species, with no obvious change in the length of the lag phase following the addition of the reducing agent (Fig. S5, A–C). This suggests that there is little to no effect on the unfolding, but only on the subsequent aggregation of the protein.

Using yADH as a substrate resulted in a slightly different chaperoning profile (Fig. 6B). In this case only ΔN11, Δ41–50 and Δ51–60 proved less effective in protecting against aggregation. Importantly however, Δ31–40 demonstrated the same increase in the ability to chaperone compared to the wild-type as in the experiments with insulin as a substrate (Fig. 6B). All the remaining constructs showed near wild-type activity. Similar to the time-dependent aggregation profiles using insulin as a substrate, no HSPB6 deletion altered the length of the lag phase before detectable yADH aggregation was observed (Fig. S5, D–F). The overall ability of HSPB6 and its deletion mutants was lower when compared to the assay with insulin. The highest protection level was approximately 70% for wild type HSPB6 against yADH, whereas it was around 90% in chaperoning insulin. It should be noted that for both substrates the ratio of substrate:sHSP was different: for insulin the highest monomer molar ratio was 1∶0.2, leading to a 5-fold molar excess of insulin, whereas for yADH this was 1∶2, leading to a 2-fold molar excess of sHSP. These ratios were based on previous publications where a 1∶1 weight ratio seems sufficient for full protection of a substrate protein [1], [24] and also empirically determined. They were chosen to have a measurable protection level at the highest ratio and a concentration dependent decrease in activity with lower ratios.

In stark contrast to the insulin and yADH chaperone assays, all constructs, including the full-length HSPB6 resulted in an increase in the final absorbance of the scattering species when incubated with reduced HEWL (Fig. 6C). Lowering the amount of the sHSP construct used in the assay led to a reduction in the absorbing species (Fig. S5, G–I) suggesting that HSPB6, and the different deletion constructs co-aggregate with the substrate. This was also confirmed by SDS-PAGE (results not shown). Even so, the constructs that showed lower protective capacity in the assays with both insulin and yADH (Δ41–50 and Δ51–60), resulted in a lower final absorbance with HEWL (Fig. 6C and Fig. S5, G–I). Similarly the more active Δ31–40 deletion construct resulted in slightly higher levels of aggregation.

With insulin as the smallest substrate used (5.8 kDa), we investigated the size of the resulting solution species 90 min after addition of DTT, with and without HSPB6, using dynamic light scattering (DLS) (Table 3). Using a single exponential fitting regime, reduced insulin alone showed a highly polydisperse peak with a hydrodynamic radius centered at 1600 nm. In the presence of both HSPB6 and the activating deletion Δ31–40, the average hydrodynamic radius of the scattering species was dramatically reduced to 28 nm and 23 nm respectively, albeit with an equivalent percentage polydispersity. Thus the data clearly show that HSPB6 inhibits the formation of large amorphous aggregates maintaining insulin in a smaller and more soluble state. These latter species though are bigger than the two HSPB6 constructs tested alone, with an estimated molecular weight of the chaperone-substrate complex in the order of a few MDa. These large polydisperse complexes are equivalent in size to those seen for the α-crystallins with the same substrate [39], [40].

**Table 3:** Size analysis by DLS of aggregated species.

Sample	Hydrodynamic radius (nm)	Polydispersity
WT^a^	4.7±1.7	36.6%
insulin + DTT^b^	1624±989.1	60.9%
insulin + WT (1∶0.2)^c^	27.7±14.6	52.5%
insulin + WT (1∶0.1)^c^	138.2±100.4	72.6%
insulin + Δ31–40 (1∶0.2)^c^	23.0±12.9	56.3%
insulin + Δ31–40 (1∶0.1)^c^	57.7±42.9	74.4%

a HSPB6 was diluted in buffer to a concentration of 1 mg/ml and the measurement was performed at 35°C.

b 0.25 mg/ml of insulin was incubated with 10 mM DTT at 37°C prior to measurement to assure full aggregation.

c 0.25 mg/ml insulin was incubated with sHSP in the indicated monomer molar ratio to yield the same concentrations as used for the chaperone assays. This mixture was incubated with 10 mM DTT at 37°C prior to measurement.

As it is the B-chain of insulin that is actually the insoluble component upon reduction [41] the average monomer weight ratio of sHSP and substrate in the soluble complex would be approximately 1∶1 assuming full binding at the 1∶0.2 insulin:sHSP ratio. These complexes would therefore be comprised of approximately 5 insulin B-chain molecules per sHSP and agree with previous observations where sHSPs seem to be capable in solubilizing their own weight [1].

In summary, there appears to be no site within the NTD that solely defines chaperoning ability, as all the truncations demonstrated some capacity to protect the tested substrates against aggregation. However, for all three substrates tested, deletion of residues 41–60 do show a marked reduction in chaperoning capacity, an observation also seen form residues 21–30 with insulin and HEWL, suggesting their possible importance in this role. Most intriguing is the increased activity of the 31–40 deletion. This region of HSPB6 is highly conserved in both human and distantly related species orthologues (Fig. 1A and Fig. S1), a finding that suggests a conserved role as a negative regulator of chaperone activity.

### Refined mapping of the 31–40 region

**Figure 7:**
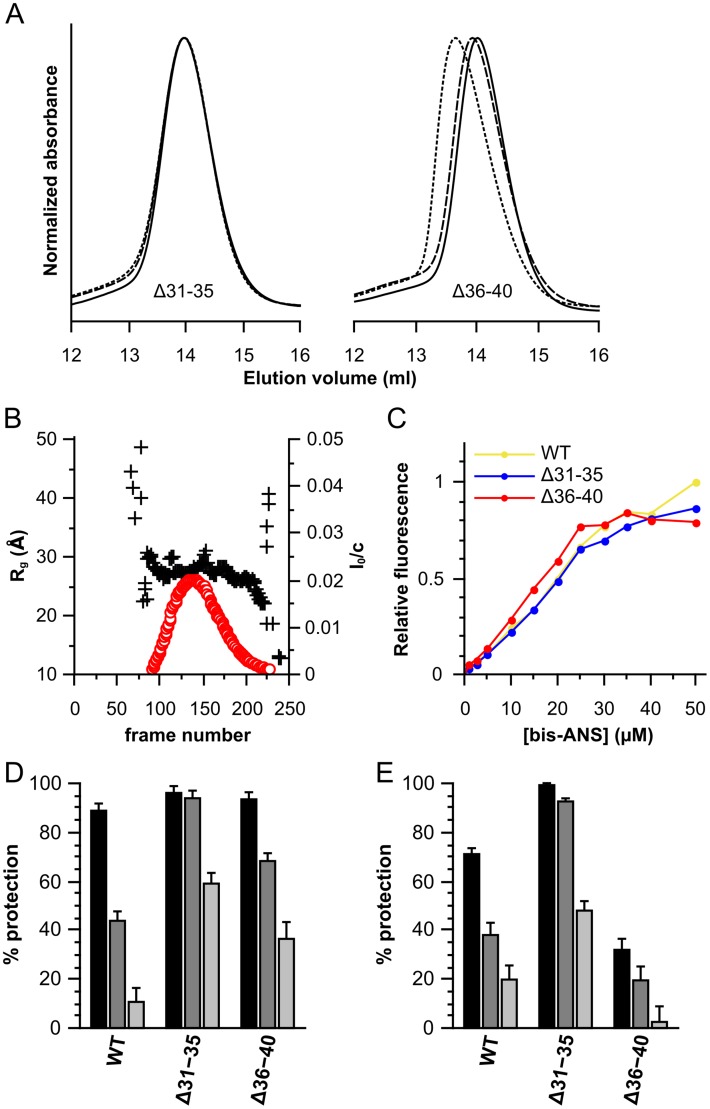
Characterization of the Δ31–35 and Δ36–40 deletion constructs of HSPB6. (A) Size-exclusion chromatography profiles, 100 µl of protein was loaded onto a Superdex 200 10/300 GL column at different concentrations. 2.5 mg/ml is shown as a solid line, 5 mg/ml as a dashed line and 10 mg/ml as a dotted line. The absorbance was normalized to the maximum for each curve. (B) SAXS analysis of Δ31–35. 250 frames were collected during elution from a Shodex KW-404F column. The radius of gyration (R_g_) and forward scattering (I_0_) were calculated at each measured point using buffer subtracted scattering curves that had been averaged by a ten-frame moving average algorithm to improve the signal-to-noise. The forward scattering values are normalized by dividing the values by the concentration of the sample at the peak maximum. The R_g_ is plotted as a black cross and scaled on the left axis, the I_0_ is shown as a red circle and scaled on the right axis. (C) Bis-ANS titration curves. 2.5 µM of protein was incubated with increasing concentrations of bis-ANS and fluorescence intensities were recorded at 490 nm with an excitation wavelength of 390 nm. HSPB6 is depicted in yellow, Δ31–35 in blue and Δ36–40 in red. (D) Chaperone-like activity using insulin as a substrate, the monomer mass molar ratios tested were 1∶0.2 (black), 1∶0.1 (dark gray) and 1∶0.05 (light gray). (E) Chaperone-like activity using yADH. The ratios used are 1∶2 (black), 1∶1 (dark gray) and 1∶0.5 (light gray).

In order to further investigate the role of residues 31–40 in defining HSPB6 chaperoning activity, two smaller five amino acid deletions (Δ31–35 and Δ36–40) were generated. Analytical SEC showed that the Δ31–35 construct mimics the behavior of the larger Δ31–40 deletion, showing ideal solution properties (Fig. 7A). Conversely Δ36–40 behaved like the wild-type protein (Fig. 7A). Again, no larger oligomeric species were observed for the two constructs at the three loaded concentrations suggesting that they are dimers in solution like the wild-type protein (data not shown). This was confirmed by SEC-coupled SAXS for the Δ31–35 construct. This deletion showed a stable R_g_ of 28 Å across the elution peak and had a calculated mass of 35 kDa (Table 2, Fig. 7B). This value is equivalent to the expected dimer (theoretical weight 33 kDa).

Bis-ANS binding studies of the two smaller deletions showed that both had a similar hydrophobicity when compared to the full-length protein (Fig. 7C). Even though the calculated hydropathy values suggested a higher hydrophobicity for the Δ31–35 construct (Table 1), we could not distinguish a clear difference with the wild type protein. This again can be due to the fact that, relative to HSPB1 and HSPB4, the NTD is solvent-exposed, thus any expected differences in hydrophobicity due to modifications of this domain will be minor when measured by this probe.

Assessment of the chaperone-like activity using insulin and yADH as substrates revealed that the region encompassing residues 31 to 35 is responsible for the negative regulatory effect seen in the larger Δ31–40 deletion (Fig. 7D and E, Fig. S6). For insulin the protection levels of both constructs were again higher than when using yADH as a substrate. Curiously, the Δ36–40 construct, which showed elevated activity when compared to wild type HSPB6 with insulin – most significantly at the highest ratio difference between the two - proved to be a worse chaperone for yADH (Fig. 7D and E). For both substrates tested Δ31–35 however showed a consistent enhancement in activity, in a similar fashion as the Δ31–40 construct, and is therefore likely the core region in negatively regulating HSPB6 chaperone activity.

### Single point mutations

Since residues 31 to 35 comprise the most conserved part of the N-terminal domain (Fig. 1A and Fig. S1), we investigated this region more thoroughly by using single point mutations. Specifically we focused on Q31 and F33, two residues with large side chains that are present in almost all sequences examined (Fig. 1A and Fig. S1). Both positions were mutated to an alanine (Q31A and F33A) and to an additional amino acid that mimicked the size of the original amino acid but altered the hydrophobicity (Q31L and F33Y). The highly conserved glycine G34 was not included in the mutational analysis, as its achiral centre and lack of side chain make this residue less amendable for choosing a suitable substitution.

**Figure 8:**
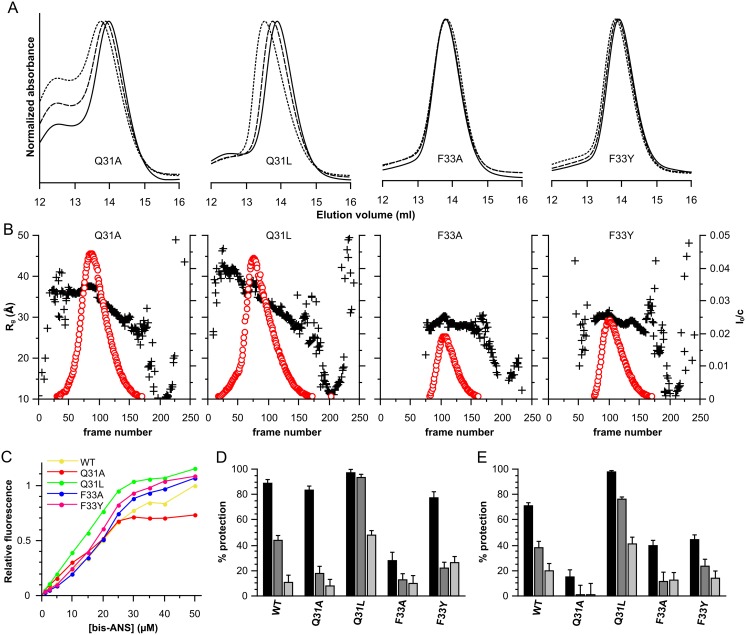
Characterization of the HSPB6 point mutations. (A) Size-exclusion chromatography profiles. 100 µl of protein was loaded onto a Superdex 200 10/300 GL column at different concentrations. 2.5 mg/ml is depicted as a full line, 5 mg/ml as a dashed line and 10 mg/ml as a dotted line. The absorbance was normalized to the maximum absorbance for each curve. (B) SAXS analysis of the HSPB6 mutants. For each construct, 250 frames were collected during elution from a Shodex KW-404F column. The radius of gyration (R_g_) and forward scattering (I_0_) were calculated at each measured point using buffer subtracted scattering curves that had been averaged by a ten-frame moving average algorithm to improve the signal-to-noise. The forward scattering values are normalized by dividing the values by the concentration of the sample at the peak maximum. The R_g_ is plotted as a black cross for each construct and scaled on the left axis, the I_0_ is shown as a red circle and scaled on the right axis. (C) Bis-ANS titration curves, 2.5 µM protein was mixed with increasing concentrations of bis-ANS and fluorescence intensities were measured at 490 nm using and excitation wavelength of 390 nm. Wild type B6 is shown in yellow, Q31A in red, Q31L in green, F33A in blue and F33Y in pink. (D) Chaperone-like activity of wild-type HSPB6 and its mutants against aggregating insulin, the monomer mass molar ratios tested were 1∶0.2 (black), 1∶0.1 (dark gray) and 1∶0.05 (light gray). (E) Chaperone-like activity of wild type B6 and its mutants against aggregating yADH. The ratios used are 1∶2 (black), 1∶1 (dark gray) and 1∶0.5 (light gray).

Analytical SEC experiments showed that both Q31 mutations had the same non-ideal behavior as seen for the wild-type protein (Fig. 8A and Table 1). Whereas Q31L had a moderately higher propensity to self-interact than the wild-type protein (Table 1), the Q31A showed the presence of a larger species, observed as a shoulder of the main peak in the elution profile (Fig. 8A). Based on column calibration this earlier eluting species of Q31A was calculated to have double the mass of the principal peak, which was equivalent to the single peak seen for the wild type. The position of the maxima of the new peak did not change with increasing loaded sample concentration, but rather an increase in relative height when compared to the later eluting species (Fig. 8A). Thus the Q31A mutant seems to assemble, in a concentration dependent manner, into what appear to be tetramers. These latter species themselves behave ideally and appear to be the upper limit of assembly with no obvious peak showing a size equivalent of the large oligomers of HSPB1, as seen for all other constructs (data not shown). In contrast to the Q31 mutants, both F33A and F33Y, showed no or limited self-association at the various concentrations tested, just like the Δ31–35 construct. The data therefore suggest that F33 is the essential residue in determining the attractive self-interaction of the wild-type protein, although the residues in the vicinity do appear to have a role in the potency of such non-ideal behavior.

When examining the SEC-coupled SAXS curves for each construct they were both similar to that of the wild-type protein (Fig. 8B and Fig. S4). However, both Q31 mutations show a bigger D_max_ of approximately 130 Å compared to 110 Å for wild-type at the same concentration. The polydispersity and associative behavior of both was further underlined by the calculated R_g_ across the peak: for Q31A this ranges from 38 to 35 Å and for Q31L from 39 to 33 Å. Using the Shodex analytical SEC column, the SAXS-based elution profile of Q31A only showed a single peak (Fig. 8B), rather than two peaks seen on the Superdex SEC column, thus the mixture of tetramers and dimers was not resolved. Nonetheless, the R_g_ range of this construct differed from the wild-type showing a stable plateau at 38 Å before the typical decrease in R_g_, likely representing the existence of two species. Both F33 mutations show a slightly smaller D_max_ of 105 Å and an R_g_ variation across the eluted peak between 30 and 28.5 Å, demonstrating the same size and ideal behavior as the Δ31–35 and Δ31–40 constructs (Fig. 8B and Fig. S4).

Bis-ANS binding studies showed minor changes in the overall hydrophobicity for F33A and F33Y, both with similar fluorescence curves as the full-length protein (Fig. 8C). The Q31A mutant demonstrated a slightly lower hydrophobicity, whereas the Q31L mutant had a slightly higher hydrophobicity compared to full length HSPB6 (Fig. 8C). The Q31A mutation appears to reach a plateau, as seen for the Δ61–70 construct, likely reflecting the propensity of this mutant to form tetramers that have an apolar environment with higher affinity for the probe. Again no large differences, as seen between full-length HSPB6, HSPB1 and HSPB4 could be observed, indicating overall the small oligomeric nature of all four HSPB6 mutants.

The chaperoning assays using reduced insulin as a substrate showed that only the Q31L mutant replicated the enhanced activity observed for the Δ31–35 and Δ31–40 constructs (Fig. 8D and E). Both Q31A and F33Y resembled the wild type protein, albeit with slightly diminished activities. Intriguingly, the F33A mutant proved to be less active at chaperoning at all concentrations tested, showing percentage protection values that were less than any of the 10 residue deletion constructs (Fig. 6A, B, Fig. S5 and S6). Similarly, the Q31L mutant showed elevated chaperone activity when tested with yADH. In contrast the three other mutants were severely hampered in their ability to protect this substrate from aggregating.

As expected from the previous assays with HEWL, all point mutations ultimately resulted in higher levels of aggregation (Fig. S6, G–H). Again, F33A the construct that showed the lowest protection for insulin and yADH, co-aggregated less with lysozyme. Surprisingly both substitutions at residue Q31 showed an increase in the lag phase prior to observable aggregation when compared to the substrate alone for the highest ratio of sHSP:substrate tested (Fig. S6, G–H). The Q31L mutant in particular afforded the most protection, increasing the lag phase by approximately 15 minutes.

**Figure 9:**
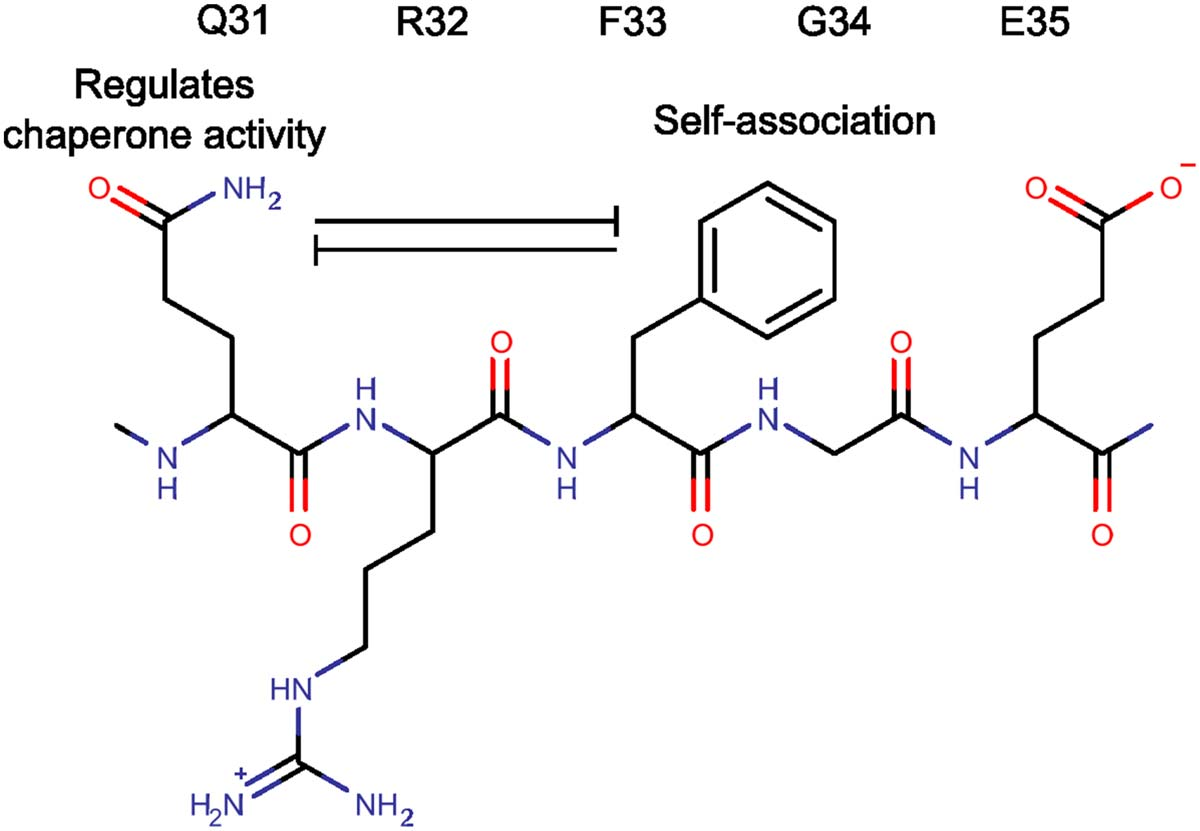
Cartoon summarizing the interplay between Q31 and F33 in defining the properties of HSPB6.

The simultaneous deletion of Q31 and F33 residues in the Δ31–35 construct enhanced chaperone-like activity but eliminated the propensity of HSPB6 to self-associate. The results from the single residue substitutions suggest that these two properties can be, in the simplest model, treated independently. Q31 appears to be the principal residue in regulating chaperone activity, while F33 can be linked to the association properties (Fig. 9).

## Discussion

Human HSPB6 belongs to a small group of sHSPs, including representatives from bacteria and plants, which appear to only exist as dimers in solution [25], [42], [43]. While being distinct with this respect from prototypical sHSP family members that are typically found as large homooligomers, these dimeric sHSPs still demonstrate equivalent chaperone-like activity [42]–[44]. This study is the first to apply a systematic approach towards characterizing the role of the N-terminal domain (NTD) in defining the chaperoning activity of a human sHSP. In vertebrate sHSPs this region has been implicated both in higher-order assembly and chaperoning [19], [21], [45]. This dual role can thus complicate interpretation of deletion or mutation analysis due to possible interfering effects. By using human HSPB6 we were able to examine the role of the NTD of this particular sHSP exclusively in terms of activity.

Previously we have shown that a number of truncations of the NTD of HSPB6, identified by limited trypsinolysis, had either reduced or no chaperone-like activity when using insulin as a substrate [25]. Expanding on this work we demonstrate here that the full NTD is essential, as complete removal of this domain prevents chaperoning of a number of standard substrates. To further delineate regions important for activity we generated 10-residue deletion mutants that covered the full N-terminal region of this sHSP. Analytical SEC and SAXS analyses of the various constructs clearly showed that small deletions in the NTD had no profound effect on the structure (Fig. 2–5). As all the deletions formed dimers in solution, similar to the full-length protein, changes in chaperone activity can thus be directly related to loss of a functional sequence rather than any change in the oligomerisation state.

None of the 10-residue deletions in the NTD resulted in a complete loss of activity seen for the ΔN truncation (Fig. 6 and Fig. S5). This result suggests that there are likely multiple, redundant substrate binding sites in this region. Even so for all substrates tested, we observed that removal of residues in the range 41 to 60 limited the chaperoning capacity of HSPB6. We additionally saw that residues 21–30 may play in role for a subset of the substrates. A similar redundancy and variability in substrate preference has also been seen for the NTDs of other sHSPs, including the dimeric HSP18.1 from *Arabidopsis thaliana*, suggesting a common mechanism [42], [46].

While the majority of truncations had a negative effect on chaperone-like activity, we unexpectedly found that deletion of residues 31–40 resulted in a strong enhancement in the capacity of HSPB6 to protect a number of substrates from forming large insoluble aggregates (Fig. 6). Further iterative deletion analysis pinpointed residues 31–35 as having a role in this phenomenon (Fig. 7 and Fig. S6). This particular region of the NTD is highly conserved, being found in five other human sHSP homologues as well as orthologues in a variety of vertebrate and invertebrate species (Fig. 1A and Fig. S1). A study where the conserved region SRLFDQFFG, corresponding to HSPB6 residues 26 to 34, was deleted in the α-crystallins also yielded an improvement in the ability to chaperone reduced insulin [47]. This deletion also resulted in smaller oligomers showing a higher turnover of the component subunits. In equivalence to these latter effects we also observed that Δ31–35 showed no weak self-association, a property readily detected by SEC with full-length HSPB6 and most other deletion constructs (Fig. 4 and Table 2). Taken together these results suggest a dual role of this region, firstly in regulating activity and secondly in stabilizing interactions between the individual dimers.

To further delineate the functional residues in the 31–35 region of HSPB6 we generated mutants of Q31 and F33, the two most highly conserved residues found present in a large range of sHSP orthologues (Fig. 1A and Fig. S1). Interestingly, no single substitution replicated all the biophysical and biochemical properties of the larger deletions suggesting the studied residues have different roles. We found that Q31 is important for chaperoning whilst F33 drives the self-association phenomenon (Fig. 8A, D and E). Moreover, our results point to an inhibitory cross-talk between these residues (Fig. 9). Both Q31 mutations resulted in a higher propensity of the HSPB6 dimers to self-associate, in particular Q31A resulted in a fraction of higher molecular weight oligomers, likely tetramers (Fig. 8A and Table 2). This suggests that Q31 somehow reduces the ‘stickiness’ of F33– possibly because of its polar nature. In contrast, both substitutions of F33 resulted in a significant reduction in chaperone activity, lower than any of the larger deletion constructs, suggesting that this residue limits the ability of Q31 to suppress activity.

Previous studies of the NTD in αB-crystallin also identified residues F24, F27 and F28 as important. Despite some discrepancies in the results, which could be due to the fact that αB-crystallin isolated from different mammalian species was used, or to differences in the assay, all studies showed either a decrease in activity and oligomeric size or a decreased stability at elevated temperatures upon mutating one or more of these residues [48]–[50]. Interestingly, the mutation of F28 in bovine αB-crystallin, which is equivalent to F33 in HSPB6 (Fig. 1A), showed decreased chaperone activity and a smaller oligomeric size at elevated temperatures [49], both properties similar to the behavior of our F33 mutants of HSPB6. A recent paper on the structure of Hsp16.0 from a fission yeast also pointed out an important role for phenylalanines in the N-terminal domain in allowing oligomer formation and chaperone-activity. In particular mutation of either F6 or F7 in this protein led to impaired chaperone function as well as disassembly of the oligomers [51] and suggests a conserved role for phenylalanines even in distantly related species.

In summary, we have extensively mapped the N-terminal domain of HSPB6, and could delineate two regions of particular functional importance. Firstly, the region between residues 41 and 60 seems to be necessary for chaperoning, as determined by light scattering using two different substrates. Secondly, we show that the highly conserved region (residues 26 to 34) includes residues responsible for the negative regulation of activity. Such repression may have a role in limiting sHSPs from aberrantly binding proteins which would have a detrimental effect on proteostasis. This study is the first full analysis, combining truncations and mutations, which confirms the importance of this region of the NTD that is conserved across different vertebrate sHSPs, and can serve as a reference for further analyses in other mammalian sHSPs. This may be particularly pertinent for understanding the activity of the disassociated species of oligomeric sHSPs, for example in the case of HSPB1 (HSP27) where phosphorylation changes the equilibrium of assembly association, biasing towards the presence of chaperone competent dimers [52].

This study also illustrates the complexity of the NTD interactions involved, as evidenced by the counter-intuitive results observed for the single residue mutations versus the larger deletions. Additionally the nature of the residue substitutions at one site had quite differing effects, where Q31L afforded the most protection to the substrates, possibly due to replacement of this polar residue by a more hydrophobic one, and Q31A had wild-type chaperone-like activity. Combined our results suggest that mutational studies of members of this chaperone family should be viewed with extra caution, as single site mutations may lead to conflicting conclusions due to a lack of understanding of the role that surrounding residues play on structure and function.

## Supporting Information

Figure S1 Multiple alignment of the N-terminal domain of sHSPs from different vertebrate species. Sequences of the N-terminal domain of human HSPB1, HSPB4, HSPB5, HSPB6 and HSPB8 were aligned against their orthologues found in bovine, rodent, Xenopus and zebrafish with Muscle [56]. Using Jalview [57] the aligned sequences were grouped based on their UniProt annotation and each group was colored using the ClustalX scheme. The conserved sequence, found present in all representative sHSPs, is highlighted beneath the alignment with a red line.
(TIF)

Figure S2 SDS-PAGE analysis of all HSPB6 constructs. Equal amounts of each purified protein were loaded on a 15% polyacrylamide gel alongside the PageRuler prestained protein ladder (Thermo Scientific). The gel was stained with Coomassie Brilliant Blue R-250.
(TIF)

Figure S3 SAXS data for all HSPB6 10-residue deletions. For all constructs the averaged scattering curve (black circles) overlayed with the regularized curve calculated from the pair-distribution function (red line), the pair-distribution function and the dimensionless Kratky plot is shown (from top to bottom, respectively). For each protein ten scattering curves around the elution maxima were scaled to the curve with the highest I_0_ and averaged using PRIMUS [58]. The intraparticle distance distribution function was solved using GNOM [59] incorporating the averaged scattering curve data up to q = 0.3 Å^−1^. The dimensionless Kratky plot of the averaged scattering data (blue circles) was generated using the reciprocal space R_g_ and I_0_ values calculated with GNOM. For reference the Guinier approximation of a spherical particle at low angles is shown as a black line using the function f(x) = x^2^*exp(−x^2^/3).
(TIF)

Figure S4 SAXS data for Δ31–35 and the single site point mutations of HSPB6. For all constructs the averaged scattering curve (black circle) overlayed with the regularized curve calculated from the pair-distribution function (red line), the pair-distribution function and the dimensionless Kratky plot is shown (from top to bottom, respectively). For each protein ten scattering curves around the elution maxima were scaled to the curve with the highest I_0_ and averaged using PRIMUS [58]. The intraparticle distance distribution function was solved using GNOM [59] incorporating the averaged scattering curve data up to q = 0.3 Å^−1^. The dimensionless Kratky plot of the averaged scattering data (blue circles) was generated using the reciprocal space R_g_ and I_0_ values calculated with GNOM. For reference the Guinier approximation of a spherical particle at low angles is shown as a black line using the function f(x) = x^2^*exp(−x^2^/3).
(TIF)

Figure S5 Chaperone activity of wild type HSPB6 and its truncations. 0.25 mg/ml of insulin (panels A–C), yADH (panels D–F) or HEWL (G–I) were incubated with HSPB6 and the 10 amino acid deletions in different ratios. Aggregation was induced by adding 10 mM DTT (final) for insulin and HEWL at 37°C or 20 mM DTT and 2 mM EDTA at 42°C for yADH. The absorbance at 340 nm was monitored for 90 min with a measurement every minute. For clarity, 5 points have been skipped for each curve. For insulin the ratios were (A) 1∶0.2; (B) 1∶0.1; (C) 1∶0.05. For yADH (D–F) and HEWL (G–I) the ratios were (D and G) 1∶2 ratio; (E and H) 1∶1 ratio; (F and I) 1∶0.5 ratio. Panels J and K represent the stability of B6 and the deletions at 37°C (J) and 42°C (K) under the same buffer conditions as used for the assay, the control experiment for ΔN is shown in dark gray with open triangles. For all plots, substrate alone is colored in black, substrate + DTT in grey, HSPB6 in yellow, ΔN11 in blue, Δ11–20 in cyan, Δ21–30 in red, Δ31–40 in green, Δ41–50 in orange, Δ51–60 in pink and Δ61–70 in violet.
(TIF)

Figure S6 Chaperone activity of wild type B6, Δ31–35, Δ36–40 and the point mutations. 0.25 mg/ml of insulin (panels A–C), yADH (panels D–F) or HEWL (G–I) were incubated with HSPB6 and its mutants in different ratios. Aggregation was induced by adding 10 mM DTT (final) for insulin and HEWL at 37°C or 20 mM DTT and 2 mM EDTA at 42°C for yADH. The absorbance at 340 nm was monitored for 90 min with a measurement every minute. For clarity, 5 points have been skipped for each curve. For insulin the ratios were (A) 1∶0.2; (B) 1∶0.1; (C) 1∶0.05. For yADH (D–F) and HEWL (G–I) the ratios were (D and G) 1∶2 ratio; (E and H) 1∶1 ratio; (F and I) 1∶0.5 ratio. Panels J and K represent the stability of B6 and the mutations at 37°C (J) and 42°C (K) under the same buffer conditions as used for the assay. For all plots, substrate alone is colored in black, substrate + DTT in grey, HSPB6 in yellow, Δ31–35 in blue, Δ36–40 in cyan, Q31A in red, Q31L in green, F33A in orange and F33Y in pink.
(TIF)

Table S1 Primers used in this work. All PCR-reactions were set up with pETHSUL-HSPB6 [55] as a template.
(PDF)
