# Supplementary figures and images for: Dissecting the Functional Role of the N-Terminal Domain of the Human Small Heat Shock Protein HSPB6
Source: PLoS One. 2014 Aug 26;9(8):e105892. doi: 10.1371/journal.pone.0105892 (PMC4144951; doi:10.1371/journal.pone.0105892)

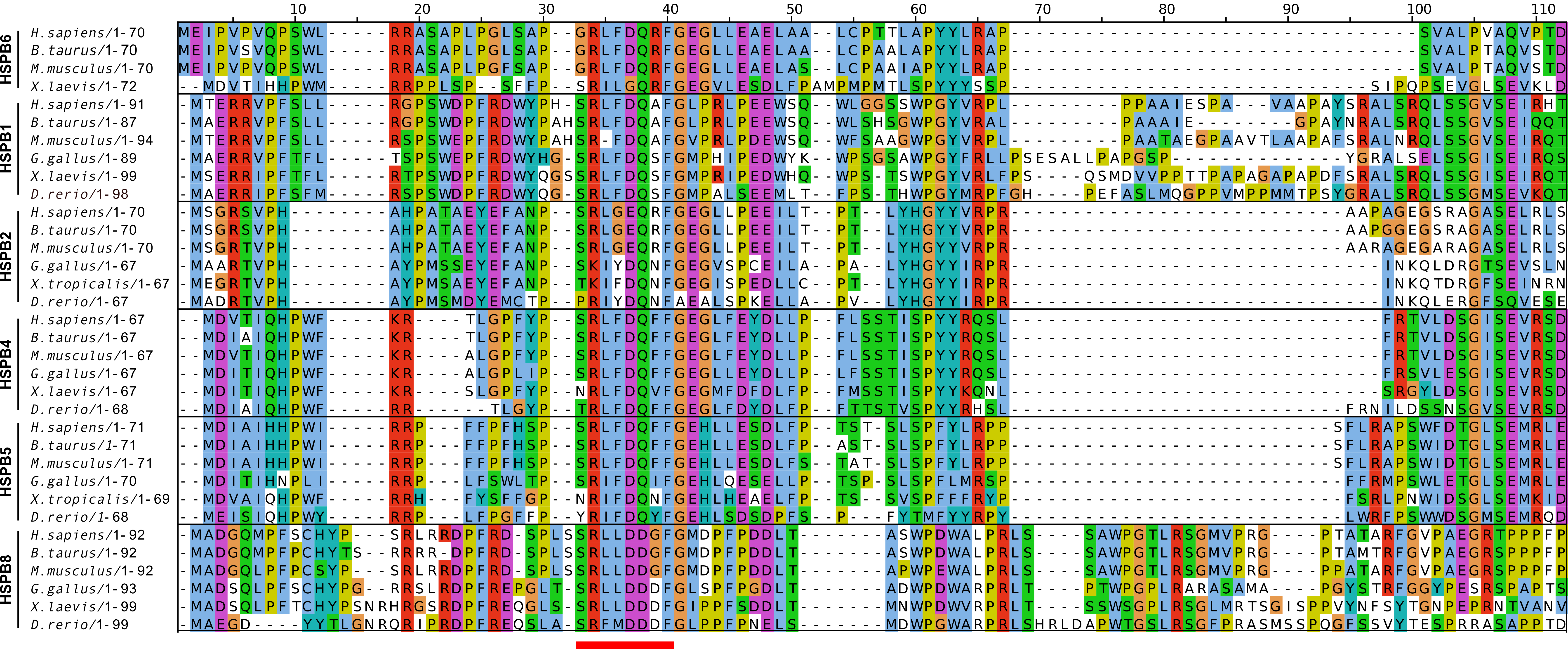

Supplement: Figure S1 — Multiple alignment of the N-terminal domain of sHSPs from different vertebrate species. Sequences of the N-terminal domain of human HSPB1, HSPB4, HSPB5, HSPB6 and HSPB8 were aligned against their orthologues found in bovine, rodent, Xenopus and zebrafish with Muscle [56]. Using Jalview [57] the aligned sequences were grouped based on their UniProt annotation and each group was colored using the ClustalX scheme. The conserved sequence, found present in all representative sHSPs, is highlighted beneath the alignment with a red line. (TIF) [file pone.0105892.s001.tif]

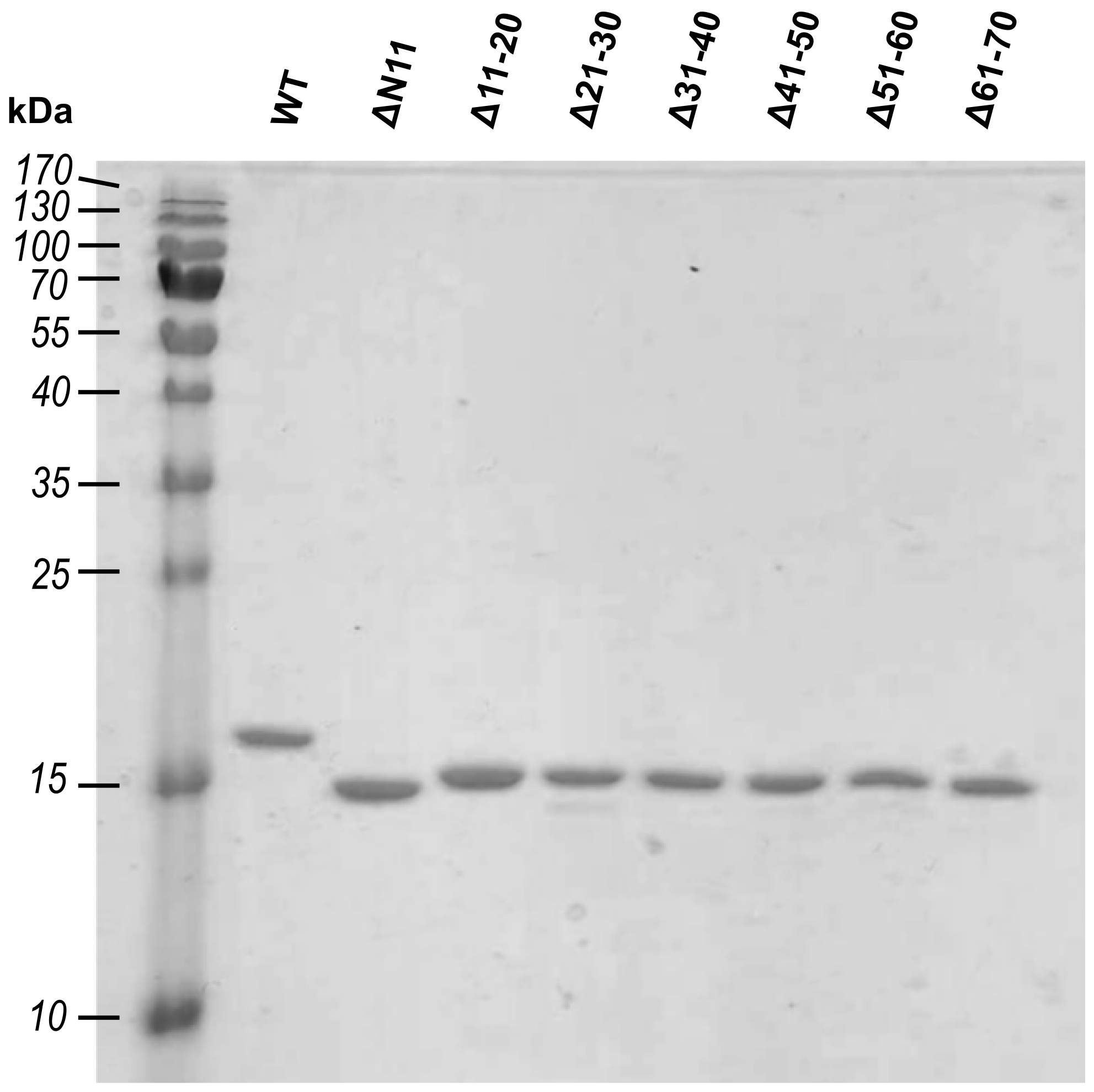

Supplement: Figure S2 — SDS-PAGE analysis of all HSPB6 constructs. Equal amounts of each purified protein were loaded on a 15% polyacrylamide gel alongside the PageRuler prestained protein ladder (Thermo Scientific). The gel was stained with Coomassie Brilliant Blue R-250. (TIF) [file pone.0105892.s002.tif]

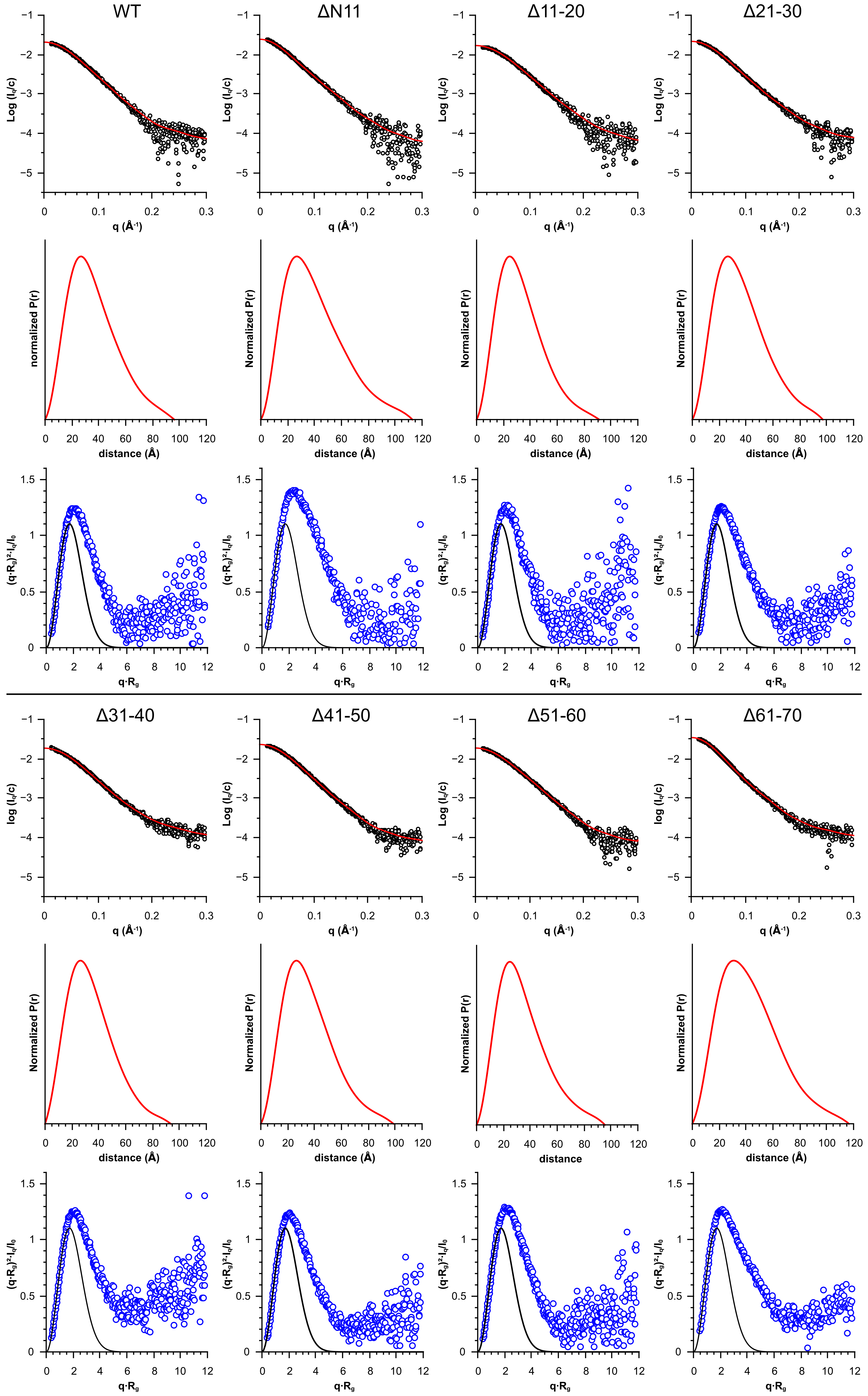

Supplement: Figure S3 — SAXS data for all HSPB6 10-residue deletions. For all constructs the averaged scattering curve (black circles) overlayed with the regularized curve calculated from the pair-distribution function (red line), the pair-distribution function and the dimensionless Kratky plot is shown (from top to bottom, respectively). For each protein ten scattering curves around the elution maxima were scaled to the curve with the highest I0 and averaged using PRIMUS [58]. The intraparticle distance distribution function was solved using GNOM [59] incorporating the averaged scattering curve data up to q = 0.3 Å−1. The dimensionless Kratky plot of the averaged scattering data (blue circles) was generated using the reciprocal space Rg and I0 values calculated with GNOM. For reference the Guinier approximation of a spherical particle at low angles is shown as a black line using the function f(x) = x2*exp(−x2/3). (TIF) [file pone.0105892.s003.tif]

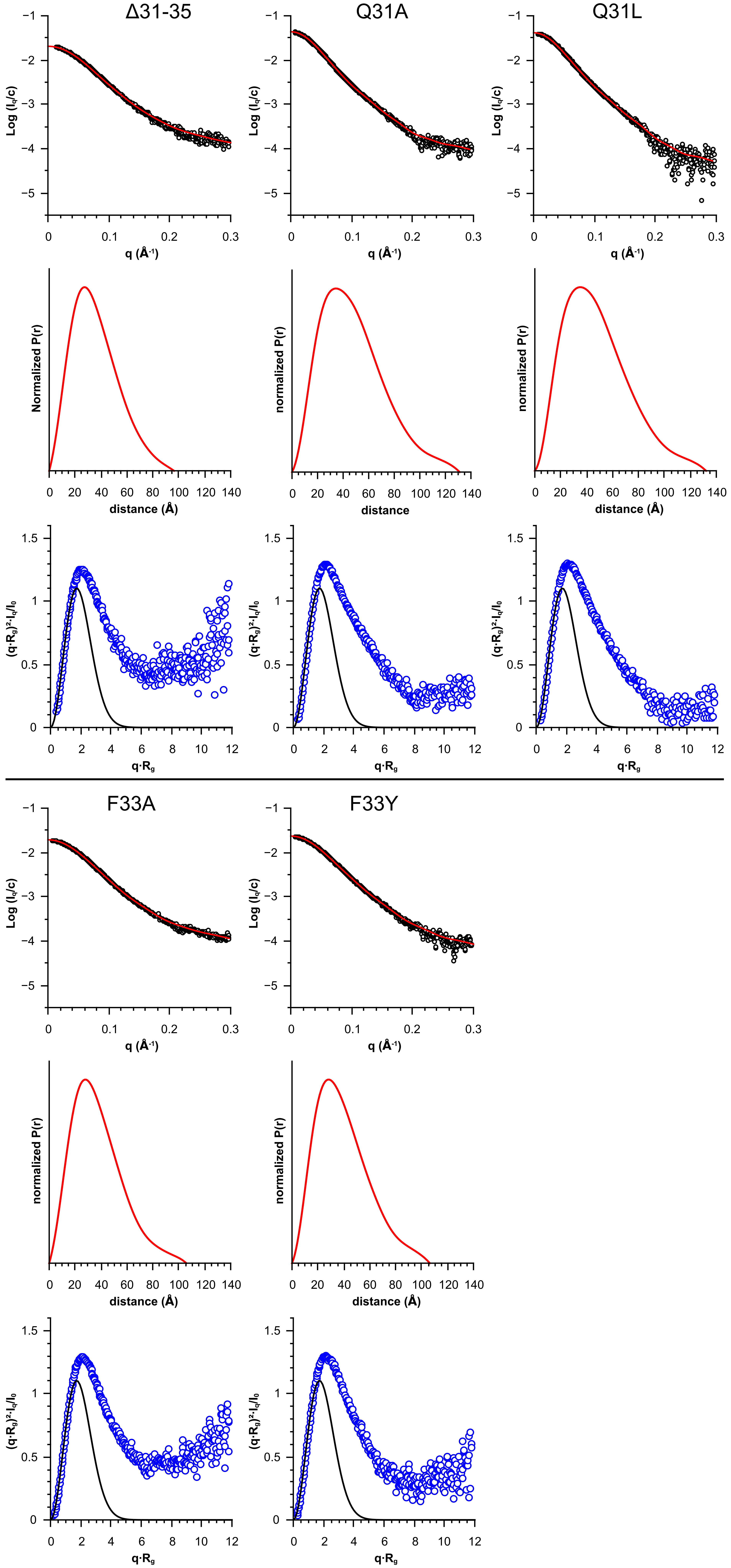

Supplement: Figure S4 — SAXS data for Δ31–35 and the single site point mutations of HSPB6. For all constructs the averaged scattering curve (black circle) overlayed with the regularized curve calculated from the pair-distribution function (red line), the pair-distribution function and the dimensionless Kratky plot is shown (from top to bottom, respectively). For each protein ten scattering curves around the elution maxima were scaled to the curve with the highest I0 and averaged using PRIMUS [58]. The intraparticle distance distribution function was solved using GNOM [59] incorporating the averaged scattering curve data up to q = 0.3 Å−1. The dimensionless Kratky plot of the averaged scattering data (blue circles) was generated using the reciprocal space Rg and I0 values calculated with GNOM. For reference the Guinier approximation of a spherical particle at low angles is shown as a black line using the function f(x) = x2*exp(−x2/3). (TIF) [file pone.0105892.s004.tif]

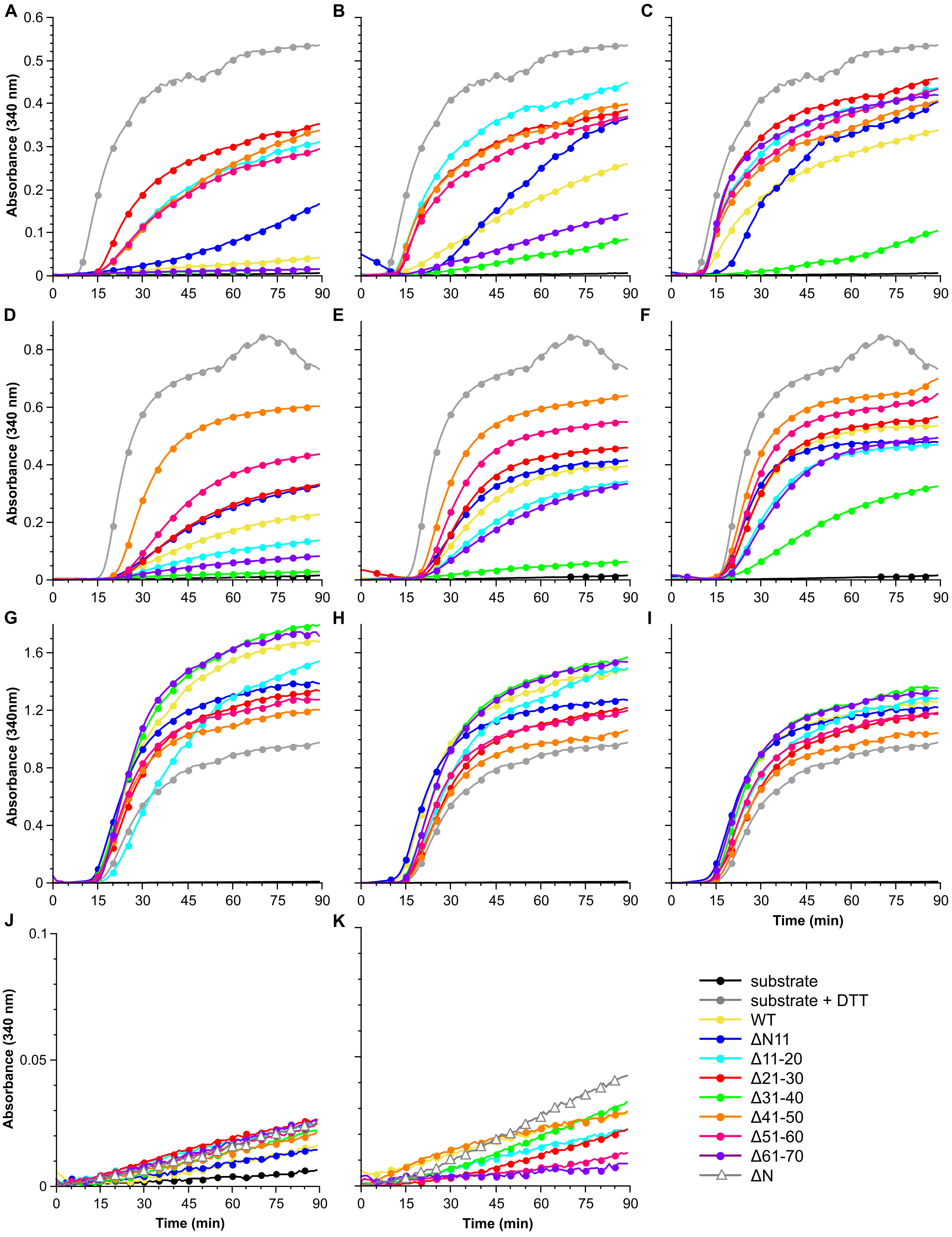

Supplement: Figure S5 — Chaperone activity of wild type HSPB6 and its truncations. 0.25 mg/ml of insulin (panels A–C), yADH (panels D–F) or HEWL (G–I) were incubated with HSPB6 and the 10 amino acid deletions in different ratios. Aggregation was induced by adding 10 mM DTT (final) for insulin and HEWL at 37°C or 20 mM DTT and 2 mM EDTA at 42°C for yADH. The absorbance at 340 nm was monitored for 90 min with a measurement every minute. For clarity, 5 points have been skipped for each curve. For insulin the ratios were (A) 1∶0.2; (B) 1∶0.1; (C) 1∶0.05. For yADH (D–F) and HEWL (G–I) the ratios were (D and G) 1∶2 ratio; (E and H) 1∶1 ratio; (F and I) 1∶0.5 ratio. Panels J and K represent the stability of B6 and the deletions at 37°C (J) and 42°C (K) under the same buffer conditions as used for the assay, the control experiment for ΔN is shown in dark gray with open triangles. For all plots, substrate alone is colored in black, substrate + DTT in grey, HSPB6 in yellow, ΔN11 in blue, Δ11–20 in cyan, Δ21–30 in red, Δ31–40 in green, Δ41–50 in orange, Δ51–60 in pink and Δ61–70 in violet. (TIF) [file pone.0105892.s005.tif]

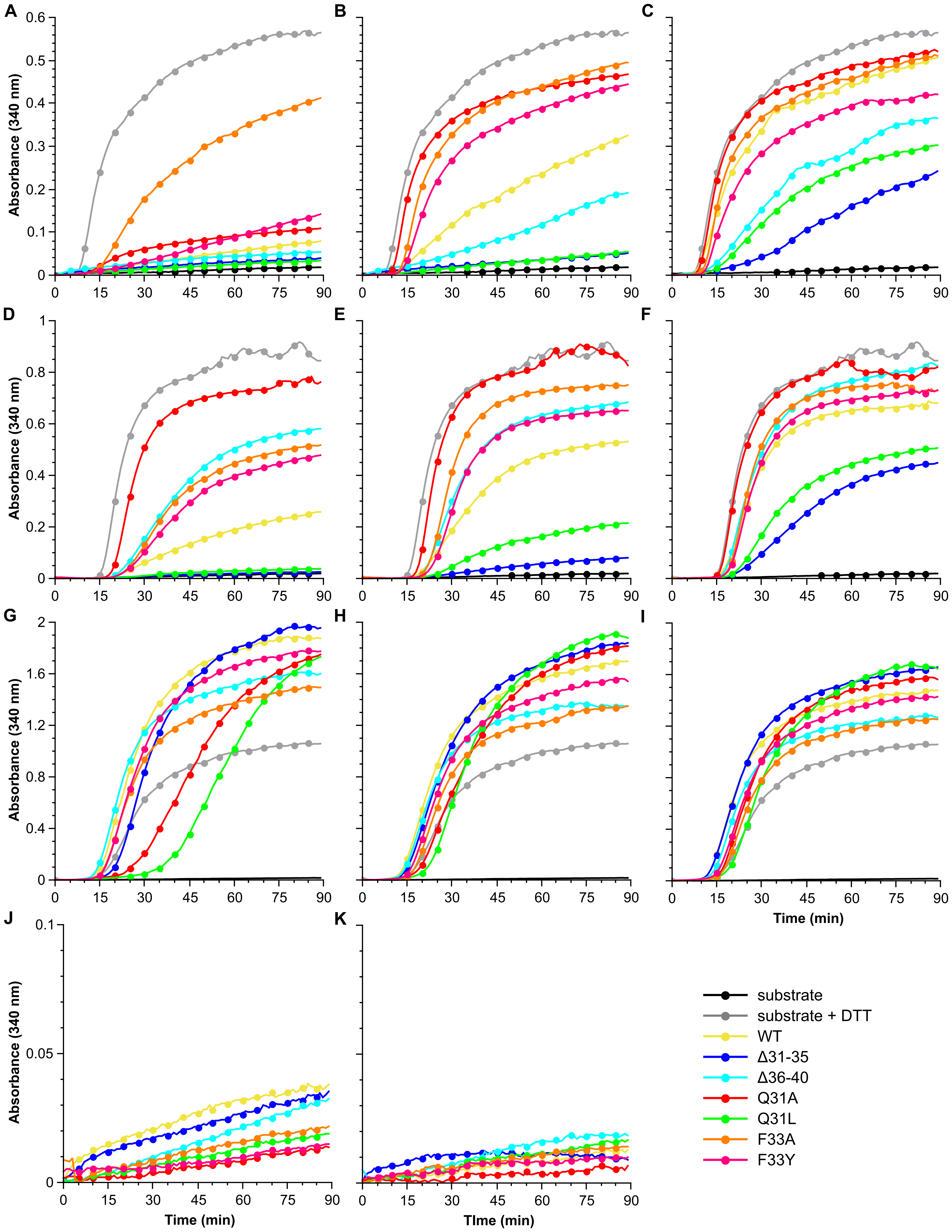

Supplement: Figure S6 — Chaperone activity of wild type B6, Δ31–35, Δ36–40 and the point mutations. 0.25 mg/ml of insulin (panels A–C), yADH (panels D–F) or HEWL (G–I) were incubated with HSPB6 and its mutants in different ratios. Aggregation was induced by adding 10 mM DTT (final) for insulin and HEWL at 37°C or 20 mM DTT and 2 mM EDTA at 42°C for yADH. The absorbance at 340 nm was monitored for 90 min with a measurement every minute. For clarity, 5 points have been skipped for each curve. For insulin the ratios were (A) 1∶0.2; (B) 1∶0.1; (C) 1∶0.05. For yADH (D–F) and HEWL (G–I) the ratios were (D and G) 1∶2 ratio; (E and H) 1∶1 ratio; (F and I) 1∶0.5 ratio. Panels J and K represent the stability of B6 and the mutations at 37°C (J) and 42°C (K) under the same buffer conditions as used for the assay. For all plots, substrate alone is colored in black, substrate + DTT in grey, HSPB6 in yellow, Δ31–35 in blue, Δ36–40 in cyan, Q31A in red, Q31L in green, F33A in orange and F33Y in pink. (TIF) [file pone.0105892.s006.tif]
