# Supplementary material for: Dissecting the Functional Role of the N-Terminal Domain of the Human Small Heat Shock Protein HSPB6
Source: PLoS One. 2014 Aug 26;9(8):e105892. doi: 10.1371/journal.pone.0105892 (PMC4144951; doi:10.1371/journal.pone.0105892)
Supplement: Table S1 — Primers used in this work. All PCR-reactions were set up with pETHSUL-HSPB6 [55] as a template. (PDF) [file pone.0105892.s007.pdf]

Construct	Direction	Primer (5' to 3')
ΔN	Forward<br>Reverse	ACCGCGAACAGATTGGTGGCGGCCACTTTTCGGTGCTGCT<br>CTTCTCGAGGAGAGTTTAGATTACTTGGCTGCGGCTGGCG
ΔN10	Forward<br>Reverse	TGGCTGCGCCGCGCCTCG<br>CGAGGCGCGGCGCAGCCAGCCACCAATCTGTTCGCGGT
ΔN11	Forward<br>Reverse	CACCGCGAACAGATTGGTGGCCTGCGCCGCGCCTCGGC<br>CTTCTCGAGGAGAGTTTAGATTACTTGGCTGCGGCTGGCG
Δ11-20	Forward<br>Reverse	GGACTTTCGGCGCCCGGAC<br>CGTCCGGGCGCCGAAAGTCCAGACGGCTGCACAGGCACAG
Δ21-30	Forward<br>Reverse	CAGCGCTTCGGCGAGGGG<br>CCCCTCGCCGAAGCGCTGGGGCAACGGGGCCGAGGC
Δ31-40	Forward<br>Reverse	GAGCTGGCTGCGCTCTGCC<br>GGCAGAGCGCAGCCAGCTCGTCAAAGAGGCGTCCGGGCG
Δ41-50	Forward<br>Reverse	GCCCCCTACTACCTGCGCG<br>CGCGCAGGTAGTAGGGGGCGGCCTCCAGCAGCCCCTC
Δ51-60	Forward<br>Reverse	GCGCTGCCCGTCGCCCAG<br>CTGGGCGACGGGCAGCGCGAGCGTGGTGGGGCAGAGC
Δ61-70	Forward<br>Reverse	CCCGGCCACTTTTCGGTGC<br>GCACCGAAAAGTGGCCGGGCACGCTGGGTGCGCGCAG
Δ31-35	Forward<br>Reverse	GGGCTGCTGGAGGCCGAG<br>CTCGGCCTCCAGCAGCCCGTCAAAGAGGCGTCCGGGC
Δ36-40	Forward<br>Reverse	GAGCTGGCTGCGCTCTGCC<br>GGCAGAGCGCAGCCAGCTCCTCGCCGAAGCGCTGGTCA
Q31A	Forward<br>Reverse	CCTCTTTGACGCGCGCTTCGGC<br>GCCGAAGCGCGCGTCAAAGAGG
Q31L	Forward<br>Reverse	CTCTTTGACCTGCGCTTCGGC<br>GCCGAAGCGCAGGTCAAAGAG
F33A	Forward<br>Reverse	GACCAGCGCGCCGGCGAGGG<br>CCCTCGCCGGCGCGCTGGTC
F33Y	Forward<br>Reverse	GACCAGCGCTACGGCGAGGG<br>CCCTCGCCGTAGCGCTGGTC
